# The skeletal muscle phenotype of the DE50-MD dog model of Duchenne muscular dystrophy

**DOI:** 10.12688/wellcomeopenres.18251.1

**Published:** 2022-09-23

**Authors:** John C.W. Hildyard, Dominique O. Riddell, Rachel C.M. Harron, Faye Rawson, Emma M.A. Foster, Claire Massey, Frances Taylor-Brown, Dominic J. Wells, Richard J. Piercy

**Affiliations:** 1 Comparative Neuromuscular Diseases Laboratory, Department of Clinical Science and Services, Royal Veterinary College, London, London, UK; 2 Langford Veterinary Services, University of Bristol, Langford, UK; 3 Cave Veterinary Specialists, George's Farm, West Buckland, UK; 4 Department of Comparative Biomedical Sciences, Royal Veterinary College, London, London, UK

**Keywords:** DMD, Duchenne Muscular Dystrophy, Animal models, Dog, DE50-MD, Biomarkers, Skeletal Muscle, Histology, Gene expression

## Abstract

**Background**: Animal models of Duchenne muscular dystrophy (DMD) are essential to study disease progression and assess efficacy of therapeutic intervention, however dystrophic mice fail to display a clinically relevant phenotype, limiting translational utility. Dystrophin-deficient dogs exhibit disease similar to humans, making them increasingly important for late-stage preclinical evaluation of candidate therapeutics. The DE50-MD canine model of DMD carries a mutation within a human ‘hotspot’ region of the dystrophin gene, amenable to exon-skipping and gene editing strategies. As part of a large natural history study of disease progression, we have characterised the DE50-MD skeletal muscle phenotype to identify parameters that could serve as efficacy biomarkers in future preclinical trials.

**Methods**: *Vastus lateralis* muscles were biopsied from a large cohort of DE50-MD dogs and healthy male littermates at 3-monthly intervals (3-18 months) for longitudinal analysis, with multiple muscles collected post-mortem to evaluate body-wide changes. Pathology was characterised quantitatively using histology and measurement of gene expression to determine statistical power and sample sizes appropriate for future work.

**Results**: DE50-MD skeletal muscle exhibits widespread degeneration/regeneration, fibrosis, atrophy and inflammation. Degenerative/inflammatory changes peak during the first year of life, while fibrotic remodelling appears more gradual. Pathology is similar in most skeletal muscles, but in the diaphragm, fibrosis is more prominent, associated with fibre splitting and pathological hypertrophy. Picrosirius red and acid phosphatase staining represent useful quantitative histological biomarkers for fibrosis and inflammation respectively, while qPCR can be used to measure regeneration ( *MYH3*, *MYH8*), fibrosis ( *COL1A1*), inflammation ( *SPP1*), and stability of DE50-MD dp427 transcripts.

**Conclusion**: The DE50-MD dog is a valuable model of DMD, with pathological features similar to young, ambulant human patients. Sample size and power calculations show that our panel of muscle biomarkers are of strong pre-clinical value, able to detect therapeutic improvements of even 25%, using trials with only six animals per group.

## Introduction

Duchenne muscular dystrophy (DMD) is a fatal X-linked muscle-wasting disease affecting approximately 1 in every 5000 new-born boys ^1^, caused by mutations in the 2.3megabase *DMD* gene. This enormous X-chromosomal gene encodes the 427kDa dystrophin protein (dp427), the core component of the dystrophin-associated glycoprotein complex (DAGC) ^2, 3^. This heteromultimeric assembly spans the sarcolemma, linking cytoskeletal actin to the extracellular matrix and buffering the myofiber membrane against the stresses of muscle contraction ^4^. In the absence of dystrophin this link is lost, leaving muscle fibres sensitive to contraction-induced damage. Dystrophic muscle thus experiences cycles of degeneration and regeneration, eventually exceeding the capacity for repair, leading to progressive loss of muscle function, and replacement of muscle tissue with fat and fibrotic scar tissue ^5, 6^. DMD patients typically require use of a wheelchair between the ages of 10 and 12, and die between the ages of 20 and 40, most commonly of respiratory or cardiac failure ^5^. Interventions such as anti-inflammatory corticosteroids and ventilation support have significantly extended patient lifespan and welfare, but these are palliative measures that do not address the primary defect ^7^: DMD remains incurable. Encouragingly, multiple therapeutic strategies aimed at restoring dystrophin protein are now under development, including some at the pre-clinical or clinical trial stage ^8– 17^. Many of these focus on exon skipping: targeting either nascent RNA or genomic DNA to elicit exclusion of one or more exons, restoring the reading frame and producing internally truncated (but functional) dystrophin protein ^18– 21^. This surge in candidate therapies places increasing demand for translational animal models, however mouse models of DMD such as the *mdx* mouse ^22, 23^ do not adequately replicate many critical aspects of human disease: dystrophin deficiency in the mouse is well-tolerated, and robust regeneration is maintained into adulthood. *Mdx* mice exhibit compensatory hypertrophy rather than muscle atrophy, and muscle fibrosis is essentially restricted to the diaphragm: these animals are consequently valuable tools for evaluating extent of dystrophin restoration, but poor models for assessment of concomitant functional efficacy ^24, 25^. Dystrophin deficient canine models such as the Golden retriever muscular dystrophy (GRMD) dog ^26^ have a more clinically relevant phenotype: dystrophic dogs exhibit many classic features of DMD, including muscle inflammation, fibrosis, and progressive weakness and atrophy, making them highly appropriate models for late-stage pre-clinical evaluation of therapeutic efficacy ^27^.

The deltaE50 muscular dystrophy dog (DE50-MD) is a new canine model of DMD ^28, 29^, carrying a mutation in the splice donor site of exon 50: this exon is consequently omitted from the mature transcript, leading to frameshift, nonsense mediated decay and absence of dystrophin protein. Crucially, the DE50-MD mutation is therapeutically tractable: skipping of exon 51 restores the reading frame ^28^. The mutation site is especially pertinent: ~50% of all human DMD mutations occur in the ‘hotspot’ between exons 45 and 54 ^30– 32^, and therapeutic strategies targeting exon 51 specifically would be of benefit to ~13% of human patients (the largest single-target cohort) ^18, 33^. The DE50-MD dog thus represents a particularly valuable pre-clinical model of DMD (indeed, this model has already been used in the first large animal study investigating CRISPR-Cas9-mediated gene editing for DMD ^34^).

As a novel canine model of DMD, it is crucial to first characterise disease in the DE50-MD dog, identifying the progression, severity and variability of pathological presentation, and concomitant functional consequences. To this end we have conducted an extensive natural history trial (Wellcome Trust grant 101550), monitoring disease progression in a large cohort of male DE50-MD dogs (and healthy male littermates) from birth to 18 months of age. Establishing a detailed timeline of emerging pathology in this manner (and the markers that best reflect this pathology) will inform pre-clinical trial design, enabling selection of sampling times, sampling methods and group sizes to construct studies that are ethically and economically sound, while remaining statistically robust. Furthermore, we have taken considerable effort to assess the dystrophic phenotype via multiple independent but complementary approaches, taking advantage of a shared core animal cohort to ensure individual studies can subsequently be directly compared even to the level of specific animals, substantially increasing the potential statistical power of disease characterisation and the ethical use of the animals (a consistent naming scheme has been adopted to aid such comparisons - see Methods). Findings from several arms of this comprehensive natural history study are already published, including measurement of blood biomarkers ^35^, skeletal muscle MRI ^36^, and cognitive testing ^37^: here we add to this growing resource by characterising the skeletal muscle phenotype in the DE50-MD dog.

Analysis of muscle samples is a critical element of disease assessment, both in DMD patients and in animal models. Immunohistochemistry (IHC) of muscle sections is the primary means of confirming a diagnosis of dystrophin deficiency ^5^, and with therapeutic approaches now achieving dystrophin restoration, measurement of muscle dystrophin mRNA (via quantitative polymerase chain reaction (qPCR)) and dystrophin protein (via IHC/western blot) is vital to establish therapeutic efficacy. Muscle histology can also be employed to assess gross pathological changes and the extent of fibrosis, fatty replacement, and inflammatory infiltration ^38^; RNA isolated from muscle tissue can be used to assess mRNA markers associated with damage and repair (such as embryonic and neonatal myosin heavy chains) ^39, 40^. For a novel canine model of DMD, establishing the extent, severity and consistency of dystrophic pathology in skeletal muscle samples is essential. We have therefore employed both longitudinal and body-wide approaches, using a large cohort of healthy and DE50-MD animals to assess dystrophic changes in the *vastus lateralis* muscle from 3–18 months of age, with multiple muscles subsequently evaluated post-mortem. Given the pre-clinical utility of the DE50-MD model, we have focussed throughout on identifying quantitative histological and gene expression markers that distinguish healthy from DE50-MD muscle, and then determining their statistical power (to aid future trial design). We reveal key age- and muscle-specific differences in skeletal muscle pathological features, but also identify biomarkers robust to such differences, and we show that a combination of longitudinal and generalised analysis is well suited to use in pre-clinical trials, likely suitable for detecting even subtle amelioration of the dystrophic phenotype with only modest animal numbers.

## Methods

### Animal husbandry

**Figure 1.**
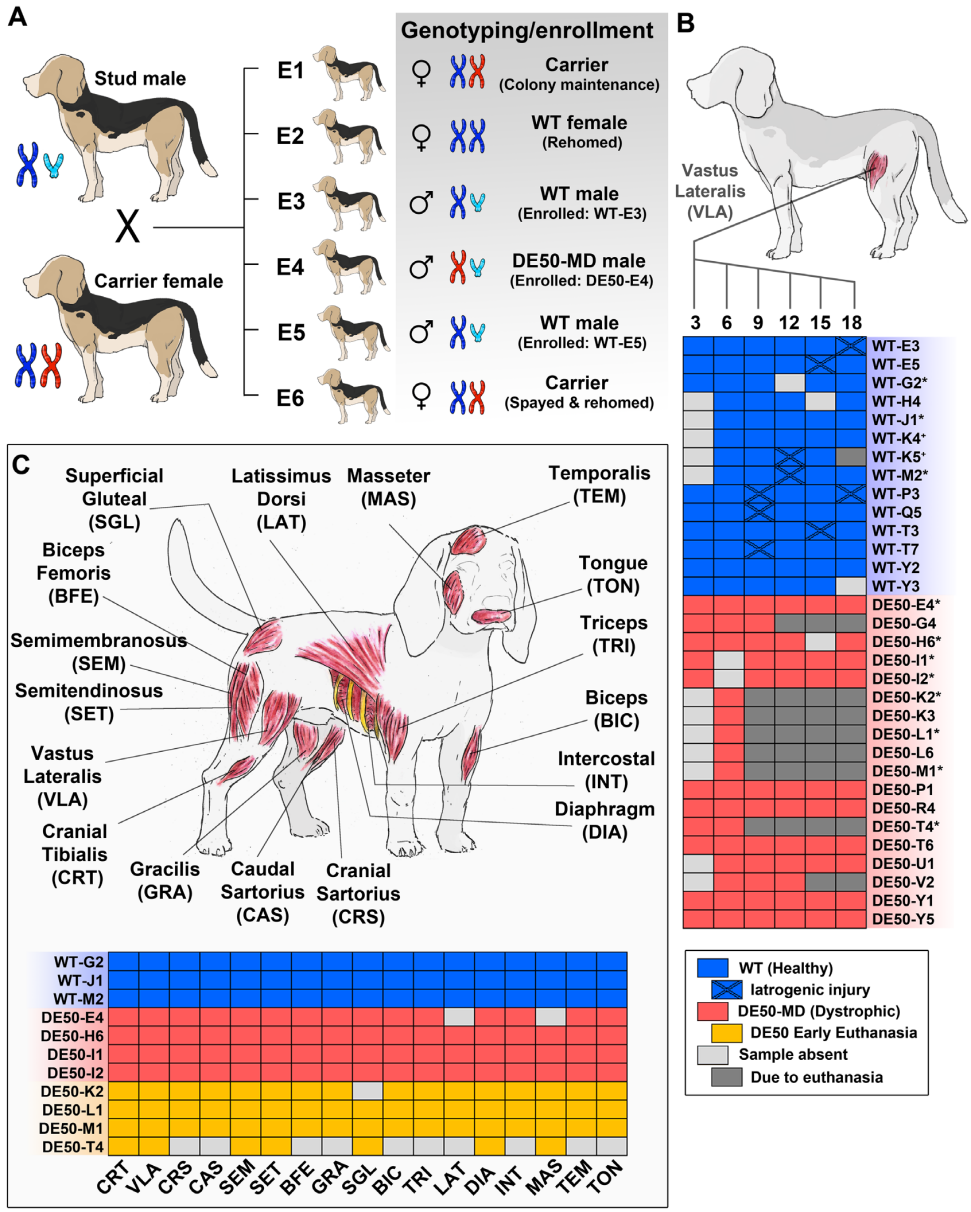
Study design and sample cohorts. Breeding, genotyping and enrolment ( **A**): carrier females are bred with healthy wild type (WT) stud males, with litters designated alphabetically. Animals within a litter are indicated numerically. Following genotyping, animals are either enrolled onto the study (DE50-MD, WT males), kept for colony maintenance (Carrier females) or rehomed (WT males, WT and carrier females (the latter after routine ovariohysterectomy)). Longitudinal analysis ( **B**): biopsy samples were collected from the left vastus lateralis muscle at 3-monthly intervals (3, 6, 9, 12, 15, 18 months) from 14 WT males and 18 DE50-MD males. 144 muscle biopsy samples were collected in total: 75 WT (3mo: N=9; 6mo: N=14; 9mo: N=14; 12mo: N=13; 15mo: N=13; 18mo: N=12) and 69 DE50-MD (3mo: N=11; 6mo: N=16; 9mo: N=12; 12mo: N=11; 15mo: N=9; 18mo: N=10). A complete summary of all individuals sampled, and the samples collected, is shown (filled blue/red squares: WT/DE50-MD samples, respectively; light grey: sample not collected/processed; dark grey: animal euthanised prior to sample collection; blue with cross: WT with evidence of iatrogenic injury). Animals subsequently used for post-mortem analysis are indicated with asterisks (*). Post-mortem ( **C**): samples were collected at 5–7 months of age (4 DE50-MD males - early euthanasia) and at 18 months of age (3 WT males, 4 DE50-MD males -humane end point) from the muscles indicated, including 9 from the pelvic limb (CRT, VLA, CRS, CAS, SEM, SET, BFE, GRA, SGL), 2 from the thoracic limb (BIC, TRI), 3 from the trunk (LAT, DIA, INT) and 3 masticatory (MAS, TEM, TON). A complete summary of all individuals sampled, and the samples collected, is shown (filled blue/red/orange squares: WT/DE50-MD/Early DE50-MD samples, respectively; light grey: sample not collected/processed). Images are created by the authors.

Dogs were housed at the Royal Veterinary College, in a dedicated canine facility with large pens, daily human interaction and access to outdoor runs and grass paddocks: conditions that exceed the minimum stipulated by the UK, Animal (Scientific Procedures) Act 1987 and according to local Animal Welfare Ethical Review Board approval. Carrier female Beagle (RCC strain, Marshall Bioresources)-cross (F3 generation) dogs derived from an original founder Bichon-Frise cross Cavalier King Charles Spaniel female carrier were mated with healthy male stud Beagles (RCC strain) to produce offspring (wild type, carrier and DE50-MD – see Figure 1A). Adult dogs were group housed (12-hour light/dark cycle; 15–24°C) until females were close to whelping; thereafter, pregnant females (singly housed) were allowed to whelp naturally and all puppies within a litter were kept with their mother in a large pen to enable nursing, with access to a bed under a heat lamp provided (~28°C). Puppies were typically microchipped at 1 week of age. From 4 weeks of age, puppies were allowed *ad lib* puppy feed (Burns) until weaning at 12 weeks, whereupon dogs not required for studies or colony maintenance were rehomed (carrier females underwent ovariohysterectomy prior to rehoming). Dogs over the age of 12 weeks received 3–4 feeds daily (2–3 feeds from 6 months of age) and *ad lib* water. All animals followed a comprehensive socialisation programme and were acclimatised to routine procedures. Welfare assessments were conducted twice daily.

### Genotyping and enrolment

All animals were genotyped within the first 2 weeks of life. Genomic DNA was prepared from cheek swabs (GeneJET genomic DNA kit, Thermofisher) and used in polymerase chain reaction (PCR) with primers spanning the splice donor site of dystrophin exon 50 (see Table 1). PCR products were purified (QIAquick, Qiagen) and submitted for Sanger sequencing (SupremeRun, Eurofins) using the forward primer only. Animals were then either rehomed (WT males and females, spayed carrier females), kept for colony maintenance (carrier females) or enrolled onto the natural history study (WT and DE50-MD: male dogs only) accordingly (see Figure 1A).

**Table 1.** PCR/qPCR primers used in this study. Nucleotide sequences of polymerase chain reaction (PCR) and quantitative PCR (qPCR) primers used, from 5’ to 3’. (*) primers used previously in 41. (†) primers used previously in 55. F and R denote forward and reverse primers respectively. Note that dystrophin isoform dp71 shares all canonical sequence from exon 63 of dp427 onward, thus exon 64 is equivalent to dp71 exon 3. Primers used for genotyping animals are indicated.

Target	Sequence (5'-3')
Dog dp427m exon 1F *	AAGGCTGCTGAAGTTGGTTG
Dog dp427m exon 2R *	TCTCTATGTGCTGCTTCCCA
Dog dp71 exon 1F *	CGGTTCTGGGAAGCTCACT
Dog dp427 exon 64R *	CCTTCTGCAGTCTTCGGAGT
Dog dp427 exon 44F *	GCGGCGGTTTCATTATGATATG
Dog dp427 exon 45R *	CAACACTTTGCCGCTGTCC
Dog dp427 exon 62F *	TCCCTGGGAGAGAGCCATC
Dog dp427 exon 64R2 *	TCATGGCAGTCCTGTAAGCT
MYH3 F	GACGCGGTCTGTCAAGGG
MYH3 R	TGCTGAGCTTTGCGGAATTT
MYH8 F	ATCAACGACCTGACAGCTCA
MYH8 R	TCTTCCTCCAGCTGACGTTT
UTRN F	AAACTTGGTGAACGCTGGAC
UTRN R	TGAAGTTGCTCGTCTGGACT
SPP1 F	GGGAGCTCTGAGGAAAAGCA
SPP1 R	GCTTCTGAGATGGGTCAGGC
MEF2C F ^†^	GCAAGCAAAATCTCCTCCCC
MEF2C R ^†^	TGGGGTAGCCAATGACTGAG
COL1A1 F	AGCCAGCAGATCGAGAACAT
COL1A1 R	ACCTGTCTCCATGTTGCAGA
Myf5 F ^†^	CGGCCTGCCTGAATGTAAC
Myf5 R ^†^	AATCCAGGTTGCTCGGAGTT
DMDseqF (genotyping)	AGCTCTGATTGGAAGGTGGT
DMDseqR (genotyping)	ACCTCAGTGTTGTGCTTTTGA

### Nomenclature

Unique animal identifiers were generated according to the following system: litters were designated alphabetically (with study enrolment commencing at litter ‘E’), with individual animals within each litter further assigned a number (thus ‘E1’ through to ‘E5’ refers to five animals from a single litter). IDs were assigned to both male and female animals, prior to genotyping. Following genotyping and enrolment, all healthy animals within the study were given the prefix ‘WT’, and all DE50-MD animals the prefix ‘DE50’: DE50-E4 and WT-E5 thus represent dystrophic and healthy littermates, respectively. To avoid confusion with conventional generation-based nomenclature (F0, F1 etc), the letter ‘F’ was not used in litter assignment (see Figure 1). All skeletal muscle samples collected for this investigation were taken as part of a large-scale longitudinal natural history study characterising disease progression at multiple levels, the findings of which are either in preparation for publication or are published ^35– 37^. Accordingly, where possible we have used the same nomenclature scheme throughout: identifiers for individual animals shown in all figures here, and where reported elsewhere, correspond to the same dogs.

### Study design

All dogs used in this study were male. Dystrophic pathology in skeletal muscle was characterised both longitudinally (single muscle, multiple time points) and body-wide (multiple muscles, single time point). For longitudinal assessment, samples (see muscle biopsy, below) were biopsied from the left *vastus lateralis* muscle at 3-monthly intervals (±2 weeks), commencing at 3 months of age until 18 months (3, 6, 9, 12, 15, 18 months, see Figure 1B). It was not possible to collect samples for all dogs at all time points (due to scheduling conflicts, time constraints, or early euthanasia – see below), but complete biopsy series were collected for 13 dogs (7 WT, 6 DE50), and in total, 144 muscle biopsy samples (75 WT, 69 DE50-MD) were collected as indicated in the schematic in Figure 1B. Note: this schematic is reproduced in full (or in part) in Figure 4– Figure 9 to indicate the precise numbers of samples and individual animals used for each investigation. Any filled boxes in Figure 4– Figure 9 should be assumed to correspond to the equivalent samples shown in Figure 1B.

For body-wide assessment, a large panel of muscles was collected post-mortem following euthanasia at either 18 months (WT, N=3, DE50-MD, N=4) or between the ages of 5 and 7 months (DE50-MD only, N=4, see below). 17 post-mortem muscles are included in this study, nine from the pelvic limb: *cranial tibialis* (CRT); *vastus lateralis* (VLA); *cranial sartorius* (CRS); *caudal sartorius* (CAS); *semimembranosus* (SEM); *semitendinosus* (SET); *biceps femoris* (BFE); *gracilis* (GRA); *superficial gluteal* (SGL), two from the thoracic limb: *biceps* (BIC); *triceps* (TRI), three from the trunk/respiratory muscles: *latissimus dorsi* (LAT); *diaphragm* (DIA); *intercostalis* (INT), and three from the masticatory muscles: *temporalis* (TEM); *masseter* (MAS); *tongue* (TON) ( Figure 1C). Not all samples were collected or processed for all individuals, but in total 174 post-mortem samples (at 18 months: 51 WT, 66 DE50-MD; at 5–7 months: 57 DE50-MD) were collected as indicated in the schematic in Figure 1C. Note: this schematic is reproduced in full (or in part) in Figure 11– Figure 14 to indicate the precise numbers of samples and individual animals used for each investigation. Any filled boxes in Figure 11– Figure 14 should be assumed to correspond to the equivalent samples shown in Figure 1C.

### Animal numbers

32 animals were used for this study (18 DE50-MD, 14 WT). All 32 were used for longitudinal muscle assessment ( Figure 1B) while 11 were also used for systemic assessment post-mortem (three 18-month-old WT: WT-G2, WT-J1, WT-M2; four 18-month-old DE50-MD: DE50-E4, DE50-H6, DE50-I1, DE50-I2; four 5–7-month-old DE50-MD: DE50-K2, DE50-L1, DE50-M1, DE50-T4 – see Figure 1C).

### ARRIVE guidelines

ARRIVE guidelines were followed for the design and conduct of the study as previously described ^35, 41^. All experimental procedures involving animals in this study were conducted according to UK legislation, within two successive project licences (70/7515 and then P9A1D1D6E) assigned under the Animal (Scientific Procedures) Act 1986 and approved by the Royal Veterinary College Animal Welfare Ethical Review Body (AWERB). All efforts were made to minimise any animal suffering throughout the study. Pre-determined endpoints for DE50-MD dogs included dehydration (unresolved by short-term fluid treatment), lethargy/motor dysfunction, weight loss/dysphagia, dyspnoea, listless behaviour/demeanour, or heart failure. Dogs were observed daily by animal technician staff and those showing any of these signs were reported to and assessed by the Study Director, the Named Veterinary Surgeon (NVS) and the Named Animal Care and Welfare Officer (NACWO). Any dogs reaching pre-determined endpoints prior to the planned 18-month study end were humanely euthanised. Euthanasia was performed using an overdose of sodium pentobarbital (250mg/kg) administered intravenously via preplaced catheter. Of the 18 DE50-MD dogs that were followed longitudinally, 10 were euthanised at the end of the planned 18-month study. 6 DE50-MD dogs (DE50-K2, -K3, -L1, -L6, -M1, -T4) were euthanised prior to 8 months of age, and another DE50-MD dog prior to 15 months of age (DE50-V2) as a result of reaching pre-determined humane endpoints related to dysphagia (none of the other pre-determined humane endpoints were encountered). 1 DE50-MD dog (DE50-G4) was euthanised at 11 months of age due to developmental elbow dysplasia, believed unrelated to the DMD phenotype ( Figure 1B). Four of the 14 WT dogs that were followed longitudinally were euthanised humanely at the end of the planned 18-month study period (WT-G2, WT-J1, WT-K4 and WT-M2), and one further WT dog (WT-K5) was euthanised at 14 months of age due to developing steroid-responsive meningitis, a condition known to affect the Beagle breed ^42^. All remaining WT dogs (9 animals) were rehomed.

### Anaesthesia

For muscle biopsy, animals were administered IV premedication (methadone 0.2mg/kg, cefuroxime 20mg/kg, medetomidine 1µg/kg, carprofen 2mg/kg) 5–10 mins prior to induction, induced with propofol (to effect: 1–4mg/kg) and maintained under sevoflurane. Animals were administered postoperative carprofen analgesia (2mg/kg) once daily for three days following muscle biopsy.

### Muscle biopsy

Muscle samples (~0.5 cm ^3^) were biopsied by open approach at 3-monthly intervals from the left *vastus lateralis* muscle, with dogs under general anaesthesia. Initial biopsy was from the proximal region of the muscle with subsequent sampling moving distal to avoid resampling previous sites (see sample evaluation, below). Muscle samples were promptly mounted on cork discs, in transverse orientation, using cryoMbed (Bright instruments Ltd) and frozen in liquid nitrogen-cooled isopentane. All muscle tissues were stored at -80°C until use.

### Sectioning

Frozen muscle tissues were cryosectioned at -25°C to 8µm thickness using an OTF5000 cryostat (Bright) and mounted on glass slides (SuperFrost, VWR). Serial sections were collected, and slides were dried at room temperature for 1 hour before storage at -80°C until use. Additional serial sections were collected in dry-ice chilled microcentrifuge tubes for RNA isolation (see below).

### Sample evaluation and inclusion criteria

Following cryosection, all *vastus lateralis* muscle biopsy samples were examined histologically (H&E -see below). Healthy muscle samples with evidence of iatrogenic injury (inadvertent resampling of previous biopsy sites) were noted. In total, 9 of the 75 WT samples (12%) showed some evidence of injury (see Figure 1B): where such injury was focal/peripheral, histological data was collected from unaffected regions. For gene expression studies (qPCR) these samples were excluded from our comparative analysis (expression data collected from these samples was in most cases consistent with injured muscle – see Longitudinal analysis: vastus lateralis gene expression and extended data, supplementary figure 2 ^43^). As dystrophic pathology precludes similar assessment in DE50-MD samples, all DE50-MD samples were included.

The 6 DE50-MD animals euthanised at 5–7 months of age (due to dysphagia) were initially assigned as ‘severe’, with dystrophic animals euthanised at 18 months of age being considered ‘moderate’. However, comparison of 6-month biopsy samples (or 5-month post-mortem samples) from ‘severe’ animals with age-matched ‘moderate’ samples revealed no significant differences in any quantitative histological or gene expression metrics except *Myf5* expression, where the difference was modest (P<0.05, Mann-Whitney U test – see extended data, supplementary figure 1 ^43^), suggesting that the dysphagic phenotype does not reflect a generalised increase in disease severity (similar results were found with serum biomarkers from the same animals ^35^). These samples were therefore included in the DE50-MD group for longitudinal analysis, and post-mortem samples from 4 of these dogs were used to assess body-wide disease at a younger age (5–7 months). 

### Muscle histology and immunofluorescence

***Haematoxylin and eosin (H&E)*.** H&E staining was conducted as described previously ^44^: mounted cryosections were allowed to equilibrate to room temperature and then immersed in Harris’ haematoxylin (diluted 1:1 in distilled water) for 3 min. Staining was regressed by dipping twice into acid alcohol, followed by blueing under running tap water for 3 min. Slides were then incubated in eosin yellowish (0.5% in water) for 3 min, washed quickly in distilled water and dehydrated through graded alcohols (70–100%). After equilibration to xylene (>1 hour) slides were mounted using DPX (solmedia).

***Picrosirius red staining (PSR)*.** PSR staining of frozen tissue used the method of Hadi *et al.* ^45^: mounted cryosections were allowed to equilibrate to room temperature before immersion in xylene for 20 min. Sections were air dried, then rehydrated via graded alcohols (100%, 90%, 80%, 70%, 5 min each) followed by distilled water, before staining with picrosirius red staining solution (0.1% Sirius red F3B in saturated (~5%) picric acid) for 45 min. Slides were washed briefly with distilled water followed by incubation in 0.5% acetic acid for 2 min to trap the stain. Slides were then dehydrated through graded alcohols as above and equilibrated to xylene (1 hour) before mounting using DPX as above.

***Acid phosphatase staining (AP)*.** Acid phosphatase staining used the acetate-buffered protocol described in 46. To prepare staining solution, 200 ml sodium acetate buffer (320mM, pH 4.8) was combined with 4 mg napthol AS-B1 phosphate (1% stock in dimethylformamide) and mixed well. Separately, 3.2 ml pararosaniline-HCl solution (4% stock in 20% conc. HCl) was mixed dropwise with 3.2 ml sodium nitrite (4% stock in distilled water, prepared fresh), incubated at room temperature for 2 mins, then added to the staining solution. pH was finally returned to 4.8 by careful addition of 1M HCl. Mounted cryosections were equilibrated to room temperature (as above) and immersed in staining solution at 37°C for 2 hours. To terminate the reaction, slides were washed well in distilled water before counterstaining for 1 min in Mayer’s haemalum (Sigma) followed by bluing under running tap water for 3 min. Slides were dehydrated rapidly through graded alcohols (~30 sec per stage) then allowed to dry completely before equilibration to xylene and mounting in DPX as above.

***Immunofluorescence (IF)*.** Immunolabelling was conducted as described previously ^47^. Slides were equilibrated to room temperature, rehydrated in phosphate-buffered saline supplemented with 0.05% Tween-20 (PBS-T) for 5 min, then incubated in blocking solution (10% goat serum in PBS-T) for 1 hour at room temperature). Both primary and secondary antibody incubations were performed for 1 hour at room temperature, with antibodies diluted in PBS-T. Where indicated, sections were subsequently stained with Hoechst (1:2000, 5 min). Slides were finally washed and mounted using Hydromount (SLS). Primary antibodies used were to perlecan (1/1000: anti-heperan sulfate clone A7L6, rat monoclonal, Sigma); dystrophin C-terminus (1/20: NCL-Dys2, mouse monoclonal, Novocastra); embryonic myosin (1/40, NCL-MHCd, mouse monoclonal, Novocastra); MEF2 (1/200, C-21, rabbit polyclonal, Santa-Cruz). Secondary antibodies were Alexa fluor conjugates (1/1000: anti-mouse Alexa488, anti-rat Alexa594, anti-rabbit Alexa594, Thermofisher).

### Imaging

Brightfield images were captured using a DM4000 upright microscope with samples illuminated using an EL6000 light source (Leica Microsystems) and a DFC550 colour camera controlled through LAS-X software (Leica). Fluorescence images were captured using a DM4000B upright microscope with samples illuminated using an EBQ100 light source and A4, L5, TX2 filter cubes (Leica Microsystems) and an AxioCam MRm monochrome camera controlled through Zen software version 3.3 (Carl Zeiss Ltd). Objectives used for both brightfield and fluorescence were 10x HC PL FLUOTAR PH1 (NA=0.3) and 20x HC PL FLUOTAR PH2 (NA=0.5). All images used can be found in the underlying data in either .tif, .czi or .zvi format ^48– 52^.

### Image analysis

Muscle fibre minimum Feret diameters (MFD) were obtained via microscopy, using images collected from muscle biopsy sections immunolabelled for perlecan, or from sections stained with Picrosirius red. Images were collected using a 10x objective (typically 3–4 per section – see underlying data ^51^). For perlecan immunofluorescence, images were analysed using either QWin (Leica) or the Fiji distribution of ImageJ (PSR-stained images were analysed using Fiji alone). In both cases the essential methodology was the same: images were subjected to binary black/white thresholding to delineate fibre perimeters, and then a watershed pass was applied to seal incompletely bounded fibre profiles (with manual correction where necessary to ensure all fibres were recognised). Fibres were designated as regions of interest (ROIs) and the appropriate parameters were extracted (MFD, cross-sectional area (CSA)). MFD values were used in preference to CSA for all analysis as MFD values are not sensitive to precise fibre orientation (CSA values can be found in the underlying data ^51^). For histograms, MFD values were assigned to 5µm bins. 800–7000 individual fibres were measured per sample: larger counts were typically obtained from younger samples, where smaller diameters result in greater numbers of fibres per imaging field. Dystrophic samples also generally yielded higher numbers (due to lower median MFD values). For violin plots, MFD outliers were identified using the ROUT algorithm ^53^ (Graphpad prism 9.3.1). All individual MFD values can be found in the underlying data ^51^.

Revertant fibres were assessed in a subset of perlecan-labelled dystrophic samples via co-immunolabelling with Dys2 and manual counting, then expressed as a percentage of total fibres per sample (as determined above). To minimise bias, imaging fields were selected randomly in the perlecan channel only prior to imaging dystrophin channel fluorescence. Consequently, many fields were negative for revertant fibres.

Quantitative analysis of picrosirius red and acid phosphatase staining was conducted using the HSB colour-space, essentially as described in 46. For each stain, 4–8 representative brightfield images per sample were captured (10x objective), and then analysed via custom-developed macros using the Fiji distribution of ImageJ. PSR staining used saturation gating to remove background, and hue to discriminate Sirius red-stained connective tissue from picric acid-stained muscle tissue (brightness was ignored). Total counts of red and yellow pixels were used to derive per-image ‘fibrosis fraction’ values (red/(red+yellow)), the arithmetic mean of which was the per-sample value (used in statistical evaluation). Acid phosphatase staining used brightness gating to remove background, saturation gating to threshold staining intensity, hue to discriminate red acid-phosphatase staining from blue/purple counterstain. As with PSR analysis, per-image ‘AP fraction’ values were derived (red/total tissue area). Given the focal nature of acid phosphatase expression in dystrophic muscle, per-sample values were expressed as the geometric mean of per-image values. Potentially batch-to-batch variation was minimised by analysing all samples from a given batch collectively, with effort taken to include both dystrophic and healthy muscles in each batch where practical.

### Gene expression

***RNA isolation*.** Total RNA was prepared from tissue cryosections using TRIzol reagent (Invitrogen) as described previously ^54– 56^: extractions were conducted essentially according to manufacturers’ protocol with the addition of a chloroform (1:1) extraction after phase separation, and inclusion of 10µg glycogen during precipitation. RNA concentrations and purity were determined via nanodrop (ND-1000, Thermofisher): samples with 260/230 ratios below 1.7 were subjected to a second isopropanol precipitation. All RNA samples were stored at -80°C until use. 

***cDNA synthesis*.** cDNA was prepared from total RNA via the RT-nanoscript 2 kit (Primerdesign/Novacyt), using 1600ng of RNA per 20µl reaction, with both oligo dT and random 9mer priming. All reactions were subsequently diluted 20-fold with DMPC-treated/nuclease-free water, to give a final cDNA concentration (assuming 1:1 conversion) of ~4ng/µl.

***qPCR*.** Quantitative PCR used SYBR green (PrecisionPLUS, Primerdesign/Novacyt). All reactions were conducted in 384-well format in duplicate or triplicate using 2µl of cDNA (~8ng) per well (10µl reaction vol), with a 3-step cycle (95°, 15sec; 60°, 20sec; 72°, 20sec) in a 384 Lightcycler (BioRad). A melt curve was included after each run: all reactions were of comparable efficiency (95–105%) and all gave a single melt peak consistent with a single amplicon. Cq values were determined via regression using CFX384 software (Biorad). Given the sample numbers, multiple plates were required for each gene: four samples were thus included on every plate to allow plate-to-plate calibration. Final per-gene Cq values were converted to relative quantities (RQ), and gene of interest (GOI) RQs were then normalised to the geometric mean of RQ values for *SDHA*, *RPL13a* and *HPRT1* (reference genes suitable for healthy and DE50-MD canine muscle, as identified previously ^55^). Primers to dystrophin (dp427 and dp71) were those used previously ^41^, as were those to *MEF2C* and *Myf5* ^55^. Primers to embryonic myosin ( *MYH3*), neonatal myosin ( *MYH8*), utrophin ( *UTRN*), Osteopontin ( *SPP1*) and collagen 1 ( *COL1A1*) were designed using primer3: all primer pairs were designed against sequence from the reference *C.familaris* genome (Ensembl), and span one or more introns. Sequences are provided in Table 1. Primers to *SDHA*, *RPL13a* and *HPRT1* were from the GeNorm *C.familiaris* primer collection (primerdesign) and sequences are thus proprietary: in accordance with MIQE guidelines ^57, 58^ we instead provide accession number, anchor nucleotide and context length, as follows: *HPRT1* (NM_001003357) Anchor nucleotide: 131, Context length 100; *SDHA* (XM_535807) Anchor nucleotide: 967, Context length 80; *RPL13A* (XM_003432726) Anchor nucleotide: 407, Context length 164. Raw Cq values can be found in the underlying data ^59^.

### Figure preparation

All figures were prepared using the Fiji distribution of ImageJ, Powerpoint 365 (Microsoft) and Photoshop CS4 (Adobe). Graphs were designed using Graphpad Prism 9.3.1, and corresponding .pzfx files can be found in the underlying data ^60^.

### Statistical analysis

Mann-Whitney U tests (comparison of early vs late euthanasia, 6-month samples only) and Pearson Correlations (revertant fibre number with age) were conducted using Graphpad Prism 9.3.1. Statistical analysis of longitudinal and post-mortem muscle parameters used a Linear Mixed Model in IBM SPSS Statistics, version 28 (open-source alternatives to Graphpad and SPSS include R, PSPP and JASP). For Mixed Model calculations, age, genotype and age/genotype (interaction) were set as fixed effects for longitudinal analysis, and muscle, group and muscle/group were used for post-mortem analysis. Post-hoc pairwise analysis (longitudinal: by age; post-mortem: by muscle) was corrected for multiple comparisons via Holm-Šídák using Graphpad Prism. Prior to analysis, log transformation was applied to data where appropriate (as determined via Shapiro-Wilk/Kolmogorov-Smirnov normality testing in Graphpad Prism). All statistics data (.sav) and statistics output (.spv) files can be found in the underlying data ^61^. Power calculation and sample size analysis used the online software resource GLIMMPSE ^62^, using a repeated measures model and either a desired statistical power of 0.8 (sample size) or a fixed group size of N=6 (power). Repeated measures for longitudinal data were animal age, while post-mortem data used individual muscles (using 10 of the 17 muscles: CRT, VLA, CRS, SEM, BFE, BIC, TRI, LAT, DIA, MAS -see Figure 1). All GLIMMPSE files (.json, .csv) can be found in the underlying data ^63^.

## Results

### Longitudinal analysis: *vastus lateralis* histology

To assess dystrophic disease progression over time, the left *vastus lateralis* muscle of healthy (WT) and dystrophic (DE50-MD) dogs was biopsied at 3-monthly intervals, from 3–18 months of age (see Figure 1 and Methods). Samples were used to prepare histological sections, with additional sections used to prepare RNA, allowing histological assessment and gene expression analysis (see below) to be conducted in parallel.

**Figure 2.**
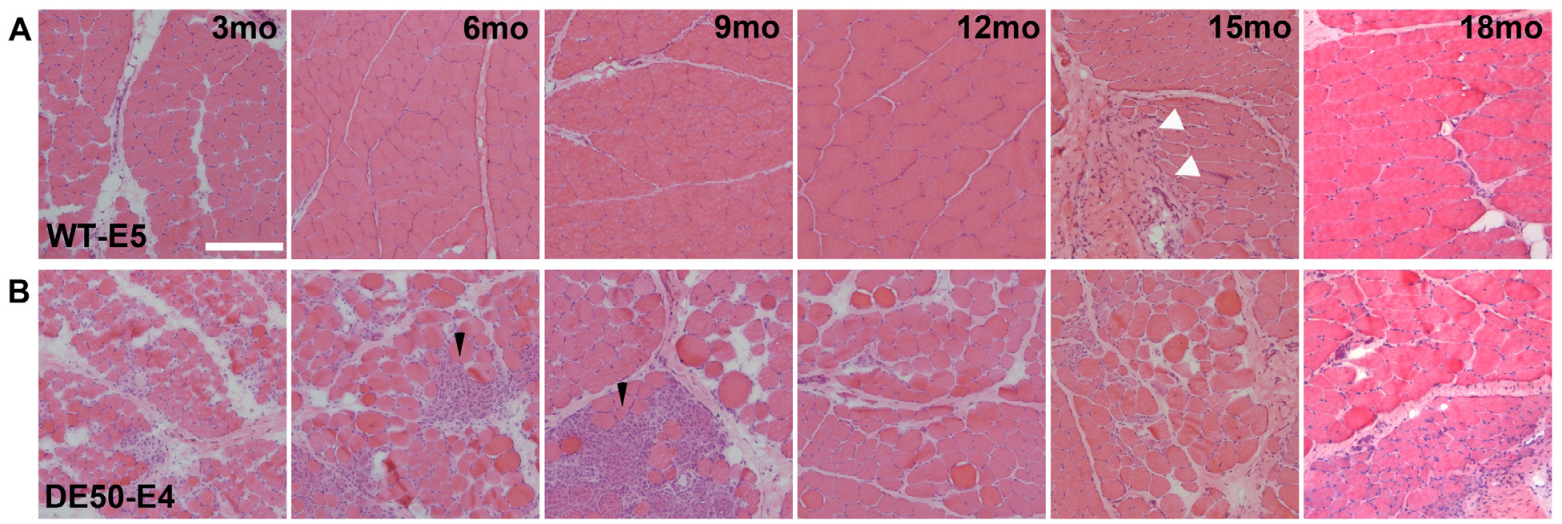
Haematoxylin and Eosin staining reveals dystrophic pathology. Representative Haematoxylin and Eosin (H&E) images of histological sections prepared from *vastus lateralis* biopsy samples. Sections shown were collected from ( **A**) one healthy animal (WT-E5), and ( **B**) one DE50-MD animal (DE50-E4) at 3, 6, 9, 12, 15 and 18 months as indicated. WT muscle shows hypertrophy associated with animal growth and permits detection of iatrogenic injury. Dystrophic muscle shows widespread disruption of fascicular architecture, hypercontractile fibres, fibre hypertrophy, focal necrosis and regeneration (most prominent at 6 and 9 months of age) and fibrotic accumulation. White arrowheads: evidence of muscle scarring -iatrogenic injury in WT-E5 15mo sample. Black arrowheads: focal degeneration/regeneration in DE50-MD muscle. Scalebar: 200µm.

We first investigated muscle pathological features histologically, via Haematoxylin and Eosin staining (H&E). Healthy muscle ( Figure 2A) demonstrated the expected hypertrophy associated with animal growth, but samples were otherwise histologically normal, with closely packed polygonal myofibres, peripherally located myonuclei and well-defined fascicular architecture. Instances of iatrogenic scarring – indicating resampling of a past biopsy site – were rare but easily identified (9 samples in 75 healthy biopsies – Figure 2A, 15mo: arrowheads), allowing these non-representative regions to be excluded accordingly (see ‘Sample evaluation’ – Methods). Staining of DE50-MD muscle ( Figure 2B) revealed characteristic pathological features associated with dystrophin deficiency. Growth-associated fibre hypertrophy was evident, but was accompanied by hypercontractile fibres, fibre necrosis and concomitant mononuclear cell infiltration, fibre regeneration (with transient internalised nucleation ^64^), marked fibre size variation (indicating both compensatory hypertrophy and regeneration), and progressive fibrosis and disruption of fascicular architecture. Acute degenerative/regenerative features were present in all DE50-MD samples but were more prominent in younger samples (3–9 months). Damage frequently presented in a focal manner, restricted to patches where multiple adjacent fibres or entire fascicles ( Figure 2B, 9mo) appeared to be at the same approximate degenerative/regenerative stage.

**Figure 3.**
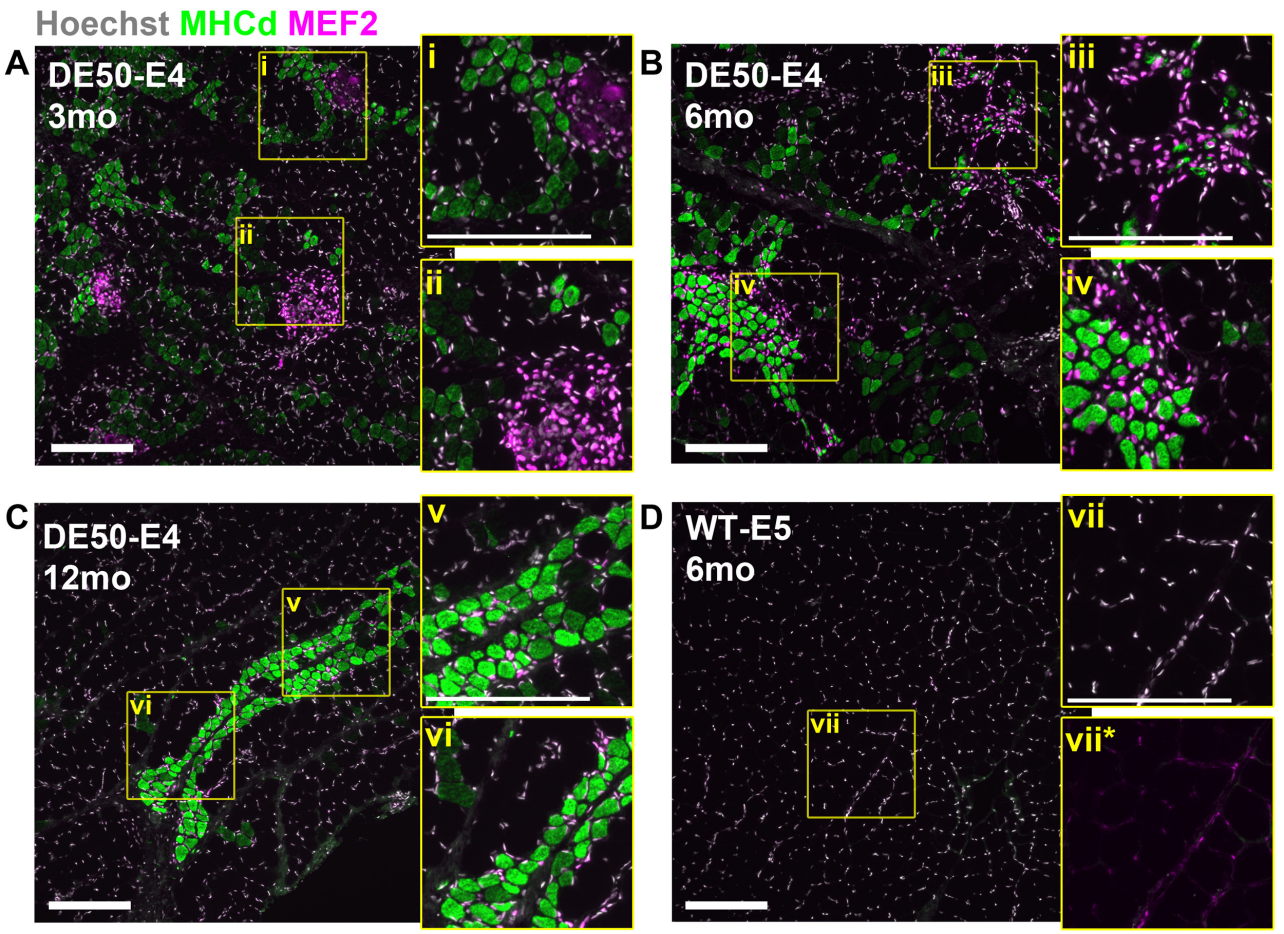
MHCd and MEF2 immunostaining reveals widespread muscle regeneration. Representative immunofluorescence (IF) images of histological sections prepared from *vastus lateralis* biopsy samples, probed with antibodies to embryonic myosin (MHCd) and to myocyte enhancer factor 2 (MEF2). Sections shown were from ( **A**– **C**) one DE50-MD animal DE50-E4 at 3, 6, 12 months and ( **D**) one WT animal WT-E4 at 6 months (as indicated). Dystrophic muscle exhibits distinct patches of regenerating (embryonic myosin positive) myofibres, associated with strongly MEF2 positive cells, and patches of MEF2 positive cells alone, while WT muscle shows low levels of MEF2 alone. Inset panels show magnified regions corresponding to regenerating myofibres with associated MEF2 positive nuclei ( **i**, **iv**, **v**, **vi**), and to richly MEF2 positive cell clusters with minimal associated dMHC staining ( **ii**, **iii**). For WT muscle, the same region is shown either magnified ( **vii**) or magnified and contrast enhanced for the MEF2 channel alone ( vii*). Scalebars: 200µm.

Histological distinction of myofibre regeneration and degeneration can be challenging via H&E alone: consequently, we next used antibodies to embryonic myosin heavy chain (NCL-MHCd) – a marker of regenerating myofibres ^40^ – and to the myocyte enhancer factor MEF2 (MEF2 C-21) -a marker of myoblast differentiation ^65^ – to more thoroughly investigate skeletal muscle regeneration in DED50-MD skeletal muscle. As expected, embryonic myosin was not detected in healthy muscle, while MEF2 was found at only low levels within myonuclei ( Figure 3D) consistent with the role of this factor in healthy muscle maintenance ^65, 66^. In dystrophic muscle ( Figure 3A–C) both markers were robustly detected, and (as suggested by H&E) muscle regeneration was indeed often focal in nature: embryonic myosin-positive fibres with MEF2-positive myonuclei were typically found in defined patches, often surrounded by cells with strongly MEF2-positive nuclei (most likely myoblasts contributing to ongoing regeneration – see Figure 3 i, iv, v, vi). Moreover, particularly in younger samples, we also observed clusters of strongly MEF2-positive cells not associated with regenerating myofibres ( Figure 3 ii) or associated with nascent myotubes rather than myofibres ( Figure 3 iii) -indicative of earlier stages in the regenerative process.

Based on these initial qualitative assessments, we identified several histological parameters that might be suited to quantitative analysis (fibre size variation, fibrosis, inflammatory infiltration).

**Figure 4.**
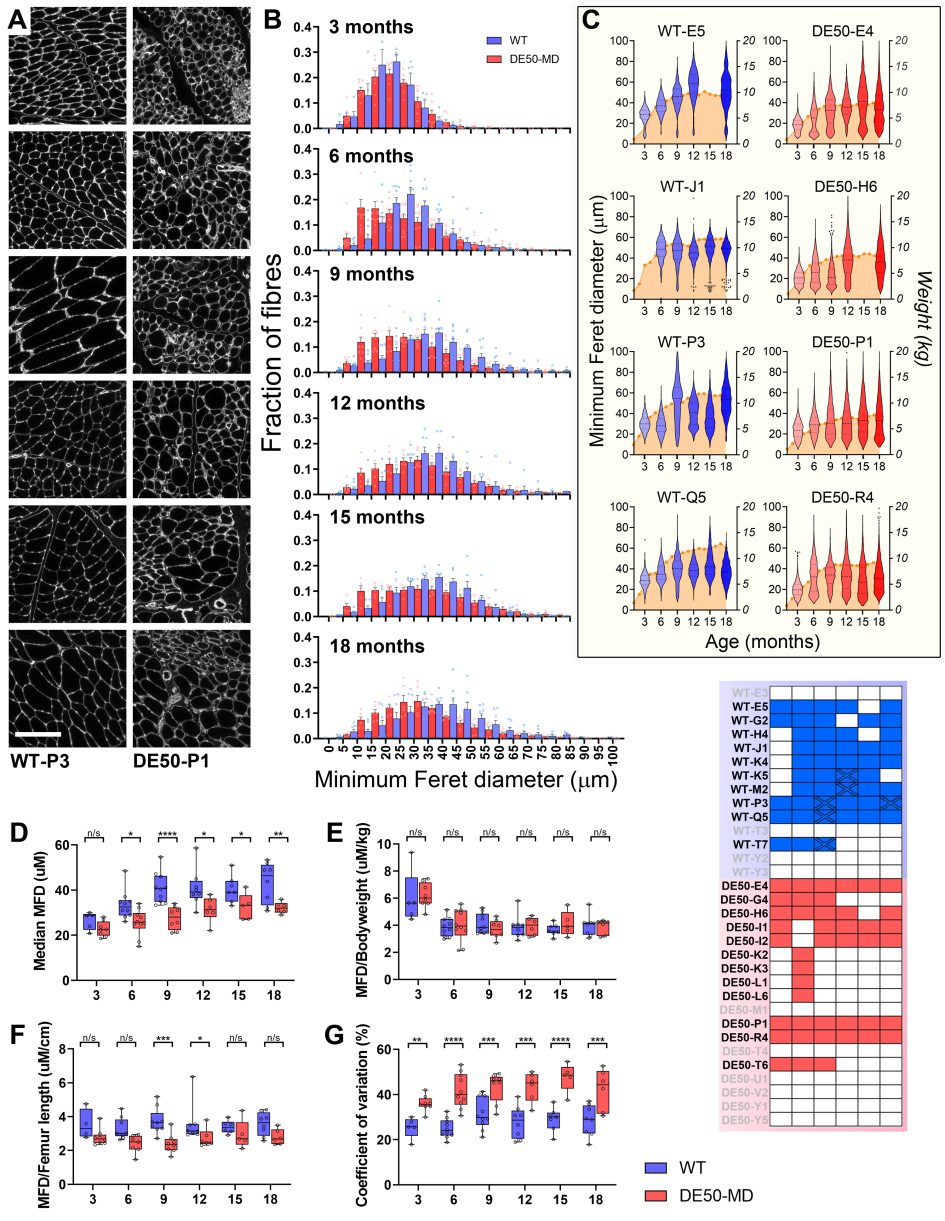
DE50-MD muscle shows marked fibre size variation. Representative immunofluorescence (IF) images of histological sections prepared from *vastus lateralis* biopsy samples, probed with an antibody to the basement membrane marker perlecan ( **A**). Sections shown were from one healthy (WT-P3) and one dystrophic (DE50-P1) animal. ( **B**) Corresponding distributions of minimum Feret diameters (MFD), for all muscle samples of the ages indicated (WT: N=5–10 samples per age; DE50-MD: N=5–10 samples per age), showing that median MFD increases with age in both genotypes, but distributions in DE50-MD muscle are typically skewed toward smaller fibres. Bars represent mean+SEM (standard error of the mean) for each bin, hollow circles indicate bin distributions for each individual. ( **C**) Violin plots of MFD values (left Y axis) aligned against corresponding bodyweights (right Y axis) for four healthy and four dystrophic animals (blue and red, respectively, shade intensity indicating increasing age), showing correlation of within-animal median MFD with growth. Note broader distributions in dystrophic animals, reflecting greater variation in fibre diameter (dots represent outlier MFD values identified via the ROUT algorithm, not included in the violin plot). Median MFD values ( **D**) increased with age as expected but were significantly lower in DE50-MD samples at all ages above 3 months (P<0.0001, Age; P<0.001, WT vs DE50; interaction, n/s). Genotype differences were eliminated following normalisation to bodyweight ( **E**) but were largely retained if normalised to femur length instead (P<0.01, WT vs DE50) ( **F**). Coefficients of variation in fibre diameters increased slightly with age and were significantly higher in DE50-MD samples at all time points (P<0.05, Age; P<0.0001, WT vs DE50; interaction, n/s) ( **G**). Post-hoc comparisons within ages are shown (Linear mixed model, Holm-Šídák correction: * = P<0.05; ** = P<0.01; *** = P<0.001; **** = P<0.0001; n/s = not significant). 48 samples from 9 WT animals and 43 samples from 12 DE50-MD animals were used for these data (a subset of our longitudinal cohort: see sample key, lower right -all filled boxes represent samples used in this analysis). Scalebar: 200µm.

***Muscle fibre profiles*.** Fibre size variation is a consequence of dystrophic damage ^38, 67^: in healthy muscle, fibre diameters are distributed over a relatively narrow and consistent range, whereas in dystrophic muscle the combination of compensatory hypertrophy, fibre splitting (where a single fibre divides longitudinally to give rise to two or more smaller fibres dwelling within the same original endomysial compartment) and regeneration of fibres can alter median fibre diameter and range -both quantitative metrics. As shown in Figure 2, fibre diameters in DE50-MD muscle varied markedly when compared with age-matched healthy controls: we quantified this phenomenon via immunolabelling for the basement membrane component perlecan ( Figure 4A), allowing measurement of fibre profiles (minimum Feret’s diameter, MFD). In both healthy and dystrophic muscle, distribution curves of MFD values mirrored animal growth ( Figure 4B), with median values increasing from 3 to 9 months of age and then remaining largely static thereafter, however median MFD was significantly lower in DE50-MD samples at all ages after 3 months ( Figure 4D), indicating greater numbers of smaller fibres. Overall prevalence of fibre splitting was rare, even in older *vastus* samples, and smaller fibres were instead usually found in patches consistent with regeneration. Considerable differences in distribution were nevertheless observed between samples, in both healthy and dystrophic muscle: partly this might reflect specific fibre type composition at each biopsy site, as data here were necessarily derived only from a representative sampling of the *vastus* (unlike comparable studies in mice where an entire muscle cross section is measured), but closer inspection suggested the principal source of variation was individual animal, with heavier animals typically exhibiting larger fibres. Indeed, distributions of fibre diameters correlated well with animal body weights ( Figure 4C) and normalising median MFD values to body weight at time of sampling essentially eliminated any differences between healthy and dystrophic samples ( Figure 4E), suggesting that median MFD might be a measurement of limited utility. Interestingly, normalisation instead to femur length (collected as part of a parallel study ^36^) which does not differ between genotypes, and which reflects animal size rather than mass, largely retained overall differences in median MFD, albeit with substantially reduced significance under post-hoc multiple comparisons ( Figure 4F). Coefficient of variation (CoV) values were a more robust metric ( Figure 4G), and here DE50-MD muscle was revealed to exhibit significantly higher variation in fibre diameter at all ages, including at 3 months (when median MFD values were comparable between genotypes).

**Figure 5.**
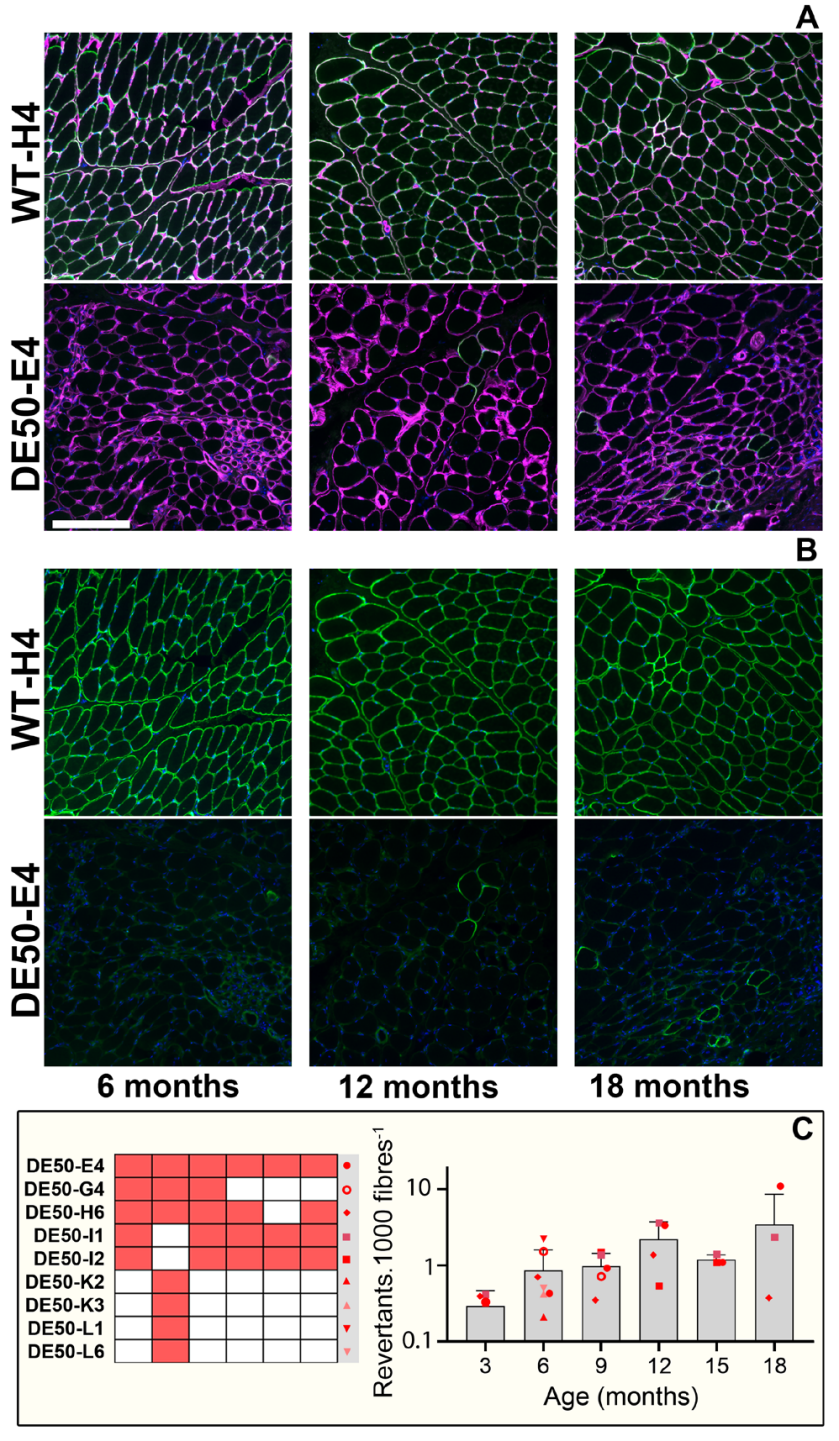
DE50-MD muscle exhibits modest numbers of revertant fibres. Representative immunofluorescence (IF) images of histological sections prepared from *vastus lateralis* biopsy samples, probed with antibodies to dystrophin (green) and perlecan (magenta) ( **A**). Sections shown were from one healthy (WT-H4) and one dystrophic (DE50-E4) animal. The same images with the dystrophin channel alone are shown in ( **B**): all WT muscle fibres are robustly labelled with dystrophin, while in DE50-MD muscle dystrophin is restricted to rare sporadic revertant fibres. Numbers of revertant fibres ( **C**) suggest a modest increase with age (Pearson correlation, P<0.01, R ^2^=0.86) but incidence remains low (~1 in 1000 fibres). Note log scale: two samples with zero detected revertants (DE50-E4 3-month, DE50-I2 18-month) are not shown. 29 samples from 9 DE50-MD animals were used for these data (sample key, lower left). Scalebar: 200µm.

**Figure 6.**
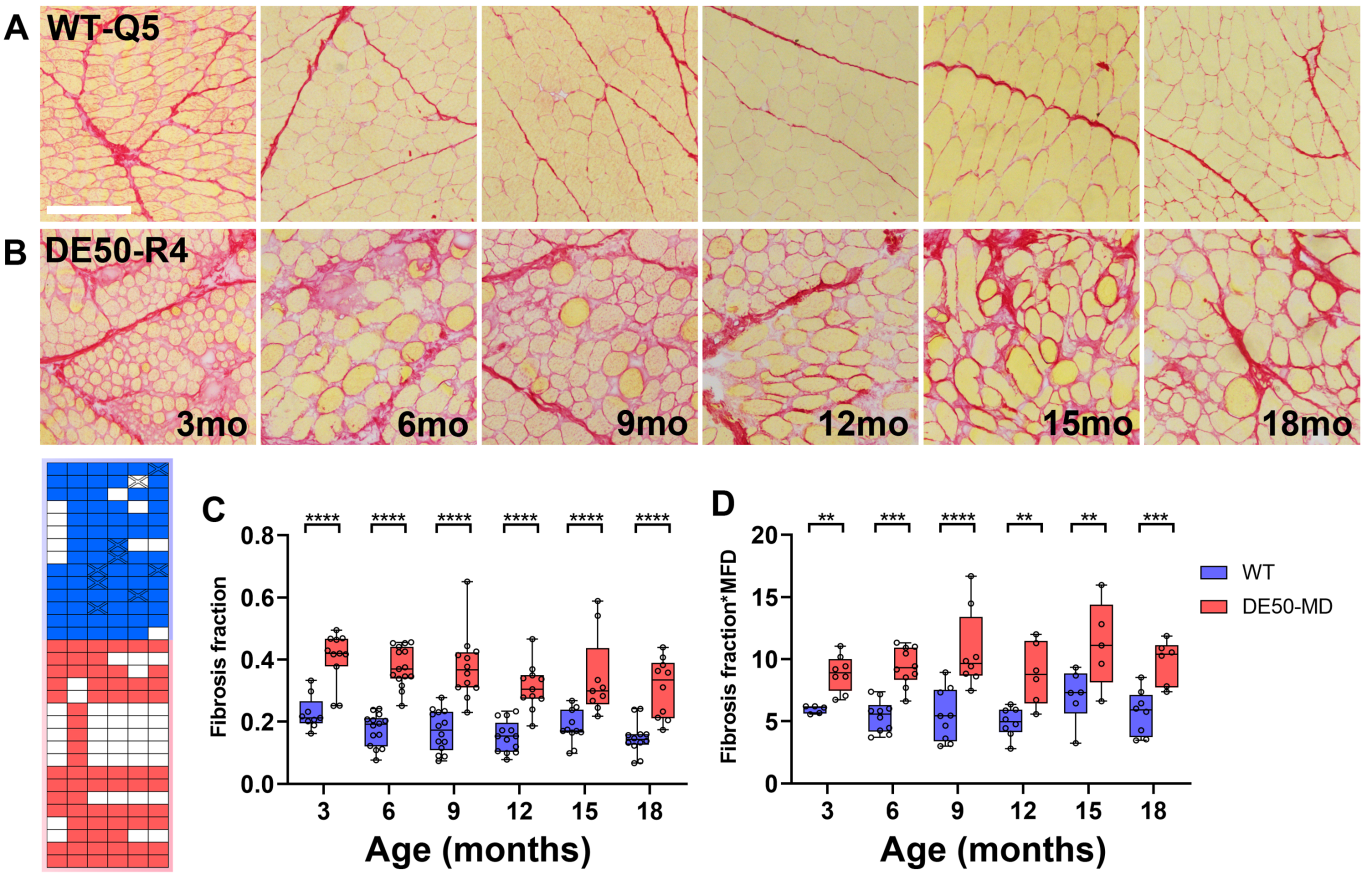
Picrosirius red staining of DE50-MD muscle reveals muscle fibrosis. Representative picrosirius red (PSR)-stained images of histological sections prepared from *vastus lateralis* biopsy samples. Sections shown were collected from ( **A**) one healthy animal (WT-Q5), and ( **B**) one DE50-MD animal (DE50-R4) at 3–18 months as indicated. Both genotypes exhibit fibre hypertrophy with increasing age, but DE50-MD muscle also shows fascicular disorganisation and increased levels of connective tissue. ( **C**) Box and whisker plots of measured fibrosis fractions (red area/total tissue area): highly significant increases were found in dystrophic muscle, alongside a modest global decline with age (P<0.0001, WT vs DE50-MD; P<0.01, Age; interaction, n/s). ( **D**) Normalising fibrosis fractions to measured median minimum Feret diameter (MFD) values (see Figure 4) retained differences between genotypes (P<0.0001, WT vs DE50-MD) but reduced effects of age (P<0.05). Post-hoc comparisons within ages are shown (Linear mixed model, Holm-Šídák correction: * = P<0.05; ** = P<0.01; *** = P<0.001; **** = P<0.0001; n/s = not significant). Each datapoint shown represents a single sample. 73 samples from 14 WT animals and 68 samples from 18 DE50-MD animals were used for these data (sample key, lower left). Scalebar: 200µm.

**Figure 7.**
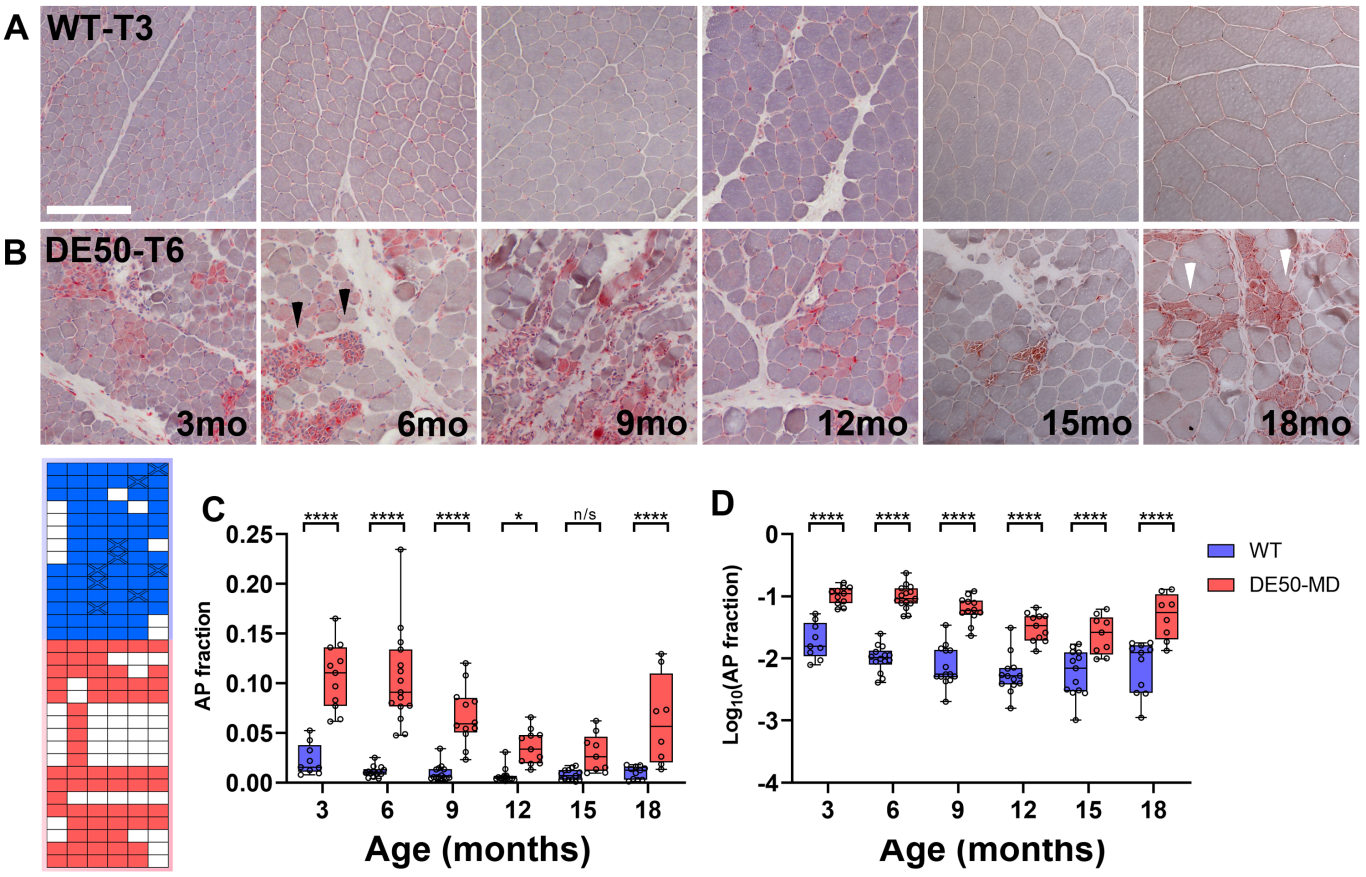
Acid phosphatase staining of DE50-MD muscle reveals pathological inflammation and remodelling. Representative acid phosphatase (AP)-stained images of histological sections prepared from *vastus lateralis* biopsy samples. Sections shown were collected from ( **A**) one healthy animal (WT-T3), and ( **B**) one DE50-MD animal (DE50-T6) at 3–18 months as indicated. In WT muscle staining appears largely restricted to capillaries. In DE50-MD muscle high levels of AP activity are found in focal regions rich in nuclei (black arrowheads), while more modest activity is found within degenerating/regenerating myofibres (white arrowheads). ( **C**) Box and whisker plots of measured AP fraction (red area/total tissue area) suggested greater AP activity in younger DE50-MD samples (3–9 months), highly significant differences between genotypes along with a significant effect of age (P<0.0001, WT vs DE50-MD; P<0.0001, Age; interaction, n/s). Analysis of log transformed data ( **D**) retained these differences while also revealing a modest interaction effect (P<0.05). Post-hoc comparisons within ages are shown (Linear mixed model, Holm-Šídák correction: * = P<0.05; ** = P<0.01; *** = P<0.001; **** = P<0.0001, n/s = not significant). Each datapoint shown represents a single sample. 74 samples from 14 WT animals and 66 samples from 18 DE50-MD animals were used for these data (sample key, lower left). Scalebar: 200µm.

**Figure 8.**
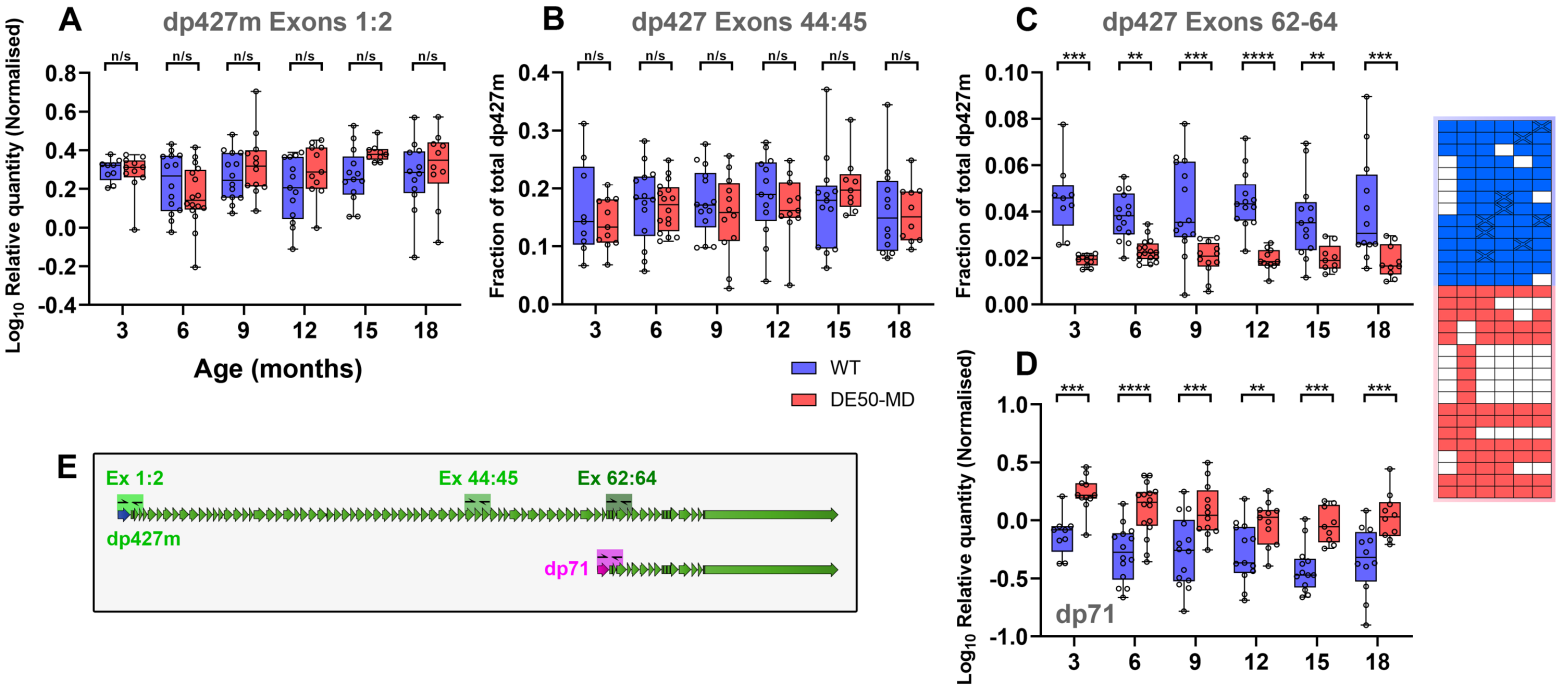
Dystrophin qPCR shows NMD of mature dp427 transcripts in DE50-MD muscle, and increases in expression of dp71. Measurement of dystrophin gene expression using quantitative PCR (qPCR) primers to the 5’ (exons 1–2), middle (exons 44–45) and 3’ (exons 62–64) regions of dp427 permits nuanced assessment of nascent/mature transcript dynamics. ( **A**) Box and whisker plots of total dystrophin mRNA (exons 1–2) revealed no differences between genotypes, suggesting transcriptional initiation remains unchanged in DE50-MD muscle. Exon 44:45 sequence ( **B**) was lower abundance as expected, but levels remained similar between genotypes, suggesting this measurement still principally reflects immature transcripts not yet subject to nonsense mediated decay (NMD). Exon 62-64 sequence ( **C**) was less abundant still (as this sequence is found predominantly in mature transcripts) but revealed marked reduction in DE50-MD samples, consistent with degradation of mature DE50 transcripts (P<0.0001, WT vs DE50-MD). Expression of the short dystrophin isoform dp71, associated with proliferating/differentiating myoblasts, was significantly higher in DE50-MD muscle ( **D**) but also declined with age in both genotypes (P<0.0001, WT vs DE50-MD; P<0.001, Age). A schematic of primer locations is shown in ( **E**). Post-hoc comparisons within ages are shown (Linear mixed model, Holm-Šídák correction: * = P<0.05; ** = P<0.01; *** = P<0.001; **** = P<0.0001; n/s = not significant). Dp427 and dp71 first exon data is shown as log10 of relative quantity (RQ) values (normalised to the geometric mean of *SDHA*, *RPL13a* and *HPRT1*), while Dp427 exon44:45 and exon 62-64 data is shown as a fraction of total dp427 (linear). Each datapoint shown represents a single sample. 75 samples from 14 WT animals and 69 samples from 18 DE50-MD animals were used for these data (see sample key, right).

**Figure 9.**
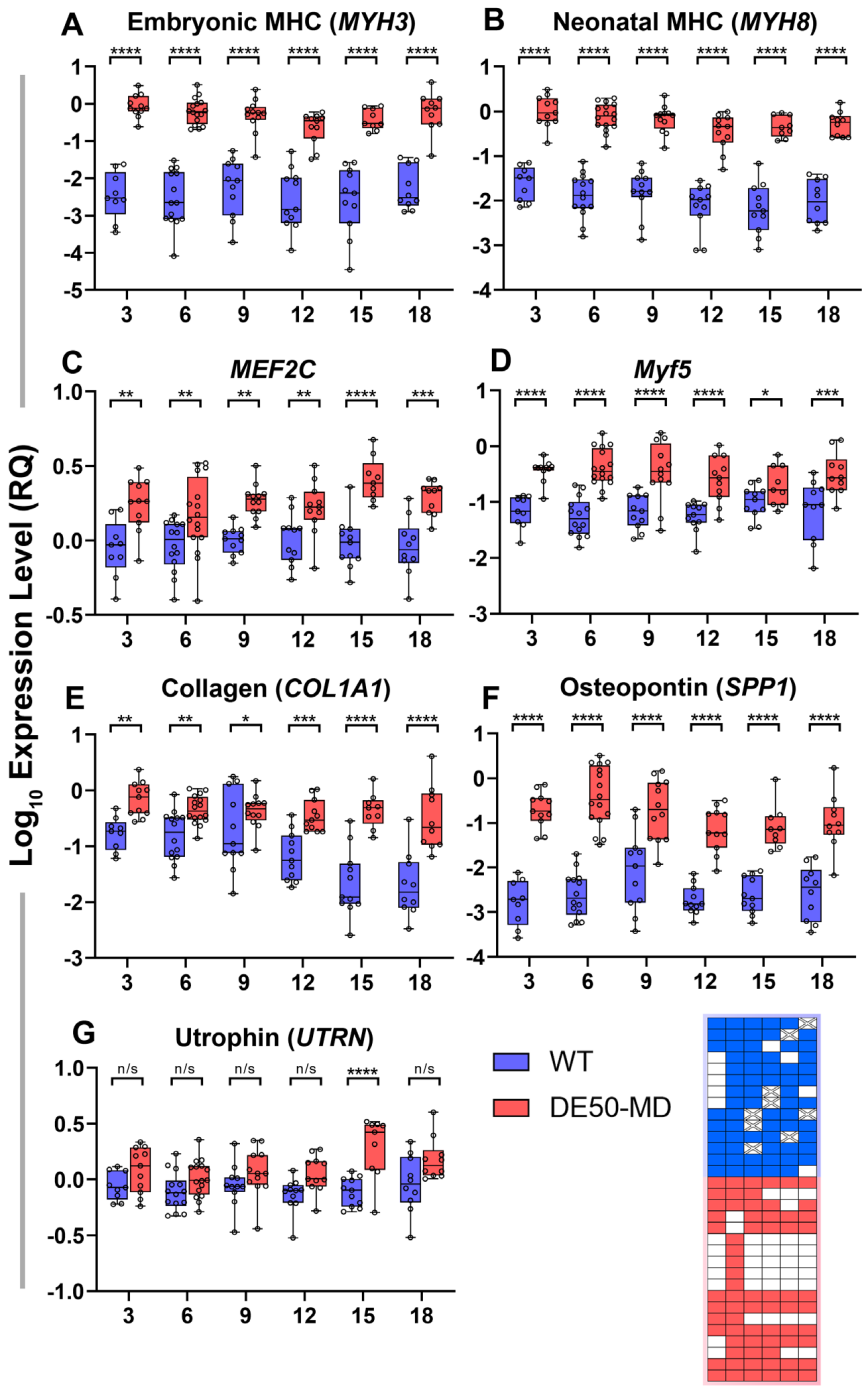
Gene expression analysis shows increases in markers of regeneration, fibrosis and inflammation in DE50-MD muscle. Box and whisker plots of gene expression using quantitative PCR (qPCR) primers to ( **A**) *MYH3* (embryonic myosin) and ( **B**) *MYH8* (neonatal myosin). *MYH8* was present at low levels in WT muscle while *MYH3* is essentially absent. Both regenerative markers were prominently elevated in DE50-MD muscle (P<0.0001, WT vs DE50-MD). *MYH8* expression further showed significant decline with age (P<0.01) in both genotypes. Expression of activated satellite cell/myoblast markers *MEF2C* ( **C**) and *Myf5* ( **D**) was also upregulated (P<0.0001, WT vs DE50-MD for both markers). Increases in *Myf5* were more pronounced in younger samples (3–9 months). ( **E**) Expression of *COL1A1* (Collagen 1) was more complex and exhibited a significant age/genotype interaction (P<0.001). While in DE50-MD muscle this biomarker remained elevated at all ages, in healthy muscle gene expression peaked at ~9 months and then subsequently declines. Greatest differences between genotypes were thus found in older samples (12–18 months). ( **F**) Expression of the inflammatory marker *SPP1* (osteopontin) was markedly elevated in dystrophic muscle (P<0.0001, WT vs DE50-MD) and while most prominently expressed in samples of 3–9 months (P<0.05, age) this marker remained elevated throughout the study period. ( **G**) *UTRN* (Utrophin) was significantly upregulated in dystrophic muscle overall (P<0.0001, WT vs DE50-MD), but differences were rarely significant within individual age categories. Statistical comparisons within ages are shown for each gene (Linear mixed model: * = P<0.05; ** = P<0.01; *** = P<0.001; **** = P<0.0001). All data is shown as log10 of relative quantity (RQ) values (normalised to the geometric mean of *SDHA*, *RPL13a* and *HPRT1*). Each datapoint shown represents a single sample. 66 samples from 14 WT animals and 69 samples from 18 DE50-MD animals were used for these data (see sample key, right).

***Revertant fibres*.** We next measured numbers of revertant fibres: sporadic fibres expressing near-normal levels of dystrophin protein (in transverse section), often found in dystrophic muscle ^68, 69^. It can be challenging to distinguish therapeutically restored dystrophin (in treatment trials) from endogenous revertant dystrophin, thus establishing the incidence of revertant fibres in any novel animal model is essential. Immunostaining for dystrophin C-terminus (using the antibody Dys2) alongside perlecan (as described above) enabled numbers of revertant fibres to be evaluated as a fraction of total fibre number. Revertant fibres were present (found in all but two of the 28 DE50-MD biopsy samples examined, Figure 5A, B), and counts suggested a modest increase with age: in older dystrophic muscle revertant patches (3 or more adjacent dystrophin-positive fibres) were also observed, albeit rarely. Overall incidence remained low and sporadic, however (typically <1 in every 1000 fibres - Figure 5C), and in most imaging fields no revertant fibres were detected, suggesting presence of revertant fibres would not preclude assessment of therapeutic dystrophin restoration.

***Muscle fibrosis: Picrosirius red*.** H&E staining suggested cumulative fibrotic remodelling of DE50-MD skeletal muscle: to measure this phenomenon we used picrosirius red (PSR) staining, revealing prominent differences between healthy ( Figure 6A) and DE50-MD ( Figure 6B) muscle samples, with widespread disorganisation of fascicular architecture and thickening of endomysial and perimysial layers. Using a high-throughput quantitative image analysis approach ^46^ we derived ‘fibrosis fraction’ values (Sirius red-stained tissue area as a proportion of total). Analysis of our sample cohort demonstrated highly significant differences between genotypes, with DE50-MD muscle exhibiting fibrosis fractions approximately double those of age-matched healthy tissue ( Figure 6C), suggesting that this metric might well be of therapeutic utility. Measured fibrosis fractions in both groups also exhibited a modest decline with increasing age, particularly over the ages of 3–12 months, a finding likely to reflect growth-associated fibre hypertrophy rather than a reduction in connective tissue *per se* (muscle tissue cross sectional area increases with the square of fibre diameter, while muscle fibre endomysium increases only linearly). Accordingly, a comparatively crude per-sample normalisation to matching median MFD values (measured previously – see Figure 4) largely eliminated this age-associated decline ( Figure 6D) while retaining significant differences between groups.

***Muscle inflammation: acid phosphatase*.** We next employed acid phosphatase (AP) staining, an enzyme-linked approach that produces a brick-red staining pattern, and which in skeletal muscle serves to label infiltrating macrophages associated with inflammation and lysosome-rich compartments found within degenerating/regenerating myofibres ^70^. We have previously shown this stain labels regions associated with inflammatory remodelling in the *cranial tibialis* muscle of 18-month-old DE50-MD dogs (manuscript under review): AP staining of our *vastus* sample cohort allowed us to assess these changes as a function of age. In healthy muscle tissue ( Figure 7A), AP staining was minimal, restricted to rare individual cells (most likely either blood cells within capillaries, or capillary endothelia). Staining in DE50-MD muscle was markedly more prominent: intense signal was found within large, cell-dense focal patches (most likely macrophage infiltrates – Figure 7B, black arrowheads) with more diffuse staining distributed throughout the sarcoplasm of specific myofibres ( Figure 7B, white arrowheads). As with PSR staining (above) we used image analysis to derive an ‘AP fraction’ -the fraction of tissue area positive for acid phosphatase activity ^46^: analysis of our longitudinal sample cohort confirmed that healthy AP fractions were close to zero as expected, while markedly higher values were found in DE50-MD muscle, particularly between 3 and 9 months of age ( Figure 7C). Image-to-image and sample-to-sample variation was however considerable (reflecting the highly focal nature of acid phosphatase activity in dystrophic muscle) and normality testing suggested a lognormal distribution for this data. Analysis of log-transformed values ( Figure 7D) accordingly showed highly significant differences at all ages, suggesting that log(AP fraction) represents a strong quantitative metric for assessment of DE50-MD muscle.

### Longitudinal analysis: *vastus lateralis* gene expression

Measurement of dystrophy-associated transcriptional changes via qPCR offers a relatively rapid, high-throughput output for disease characterisation, and is moreover immediately quantitative, facilitating statistical assessment and associated potential therapeutic evaluation. This approach further allows investigation of parameters not necessarily available at the protein level, such as expression of dystrophin (which is unconventional ^2, 41, 71, 72^). Using our longitudinal *vastus lateralis* sample collection and building on the insights gained from histological studies (above) we thus investigated transcriptional changes associated with dystrophic pathology in the DE50-MD dog. Use of RNA isolated from sections serial to those used for histology (above) further permits qPCR data to be directly evaluated against equivalent per-sample histological parameters (and vice versa).

***Dystrophin*.** We first measured expression of muscle dystrophin mRNA, using a multi-site strategy we have employed previously ^41, 47^, targeting exons 1:2, 44:45 and 62-64 of dp427m ( Figure 8E). The ~16-hour transcription time required for full-length dystrophin (with co-transcriptional splicing ^71^) means spliced exon 1:2 sequence arises early and persists throughout transcript lifespan, while exon 62–64 sequence arises only shortly before transcript completion: using primers directed to these regions effectively allows changes in transcriptional initiation (nascent dp427) to be distinguished from changes in transcript stability (mature dp427). This distinction is relevant, as we and others have previously shown that most dystrophin mRNA even within healthy skeletal muscle is nascent: mature full-length dp427 transcripts comprise only a minority fraction ^41, 47, 72^. While the frameshift and concomitant premature termination codon (PTC) generated by omission of exon 50 should elicit degradation of dp427 mRNA via nonsense mediated decay (NMD), this occurs only following nuclear export ^73^: mature mRNAs are subject to NMD, but immature transcripts are not. Previous work (using a subset of the sample cohort used here) suggested that nascent dp427 expression was comparable between healthy and DE50-MD animals ^41^. The data here strongly supports this ( Figure 8A): no differences were found in levels of nascent dystrophin mRNA between healthy and dystrophic muscle at any age, and nor did expression within a genotype change with age. Exon 44:45 and 62–64 sequences arise ~8 hours and ~1 hour before transcript completion, respectively, and consequently each represents a subset of total dp427 (the latter most closely reflecting fully mature transcripts). Normalising to exon 1:2 (total) expression thus provides approximate relative fractions of these regions. Exon 44:45 sequence comprised a modest fraction of total, but was not significantly different between genotypes, again showing that nascent (pre-NMD checkpoint) transcripts are in the majority ( Figure 8B). Exon 62–64 sequence in contrast was markedly reduced in DE50-MD samples at all ages ( Figure 8C), confirming that mature transcript losses to NMD can be detected, and that this metric might serve as a means of assessing therapeutic restoration of transcript stability.

We further measured expression of the short dystrophin isoform dp71, targeting its unique first exon sequence ( Figure 8E) to distinguish this isoform from dp427 transcripts (and vice versa) which otherwise share all sequence from exon 63 onward ^2^. Dp71 is expressed in blood vessel endothelia ^74^ (and is thus present at low levels within skeletal muscle) but is also found in proliferating myoblasts ^75– 77^, and thus might serve as a marker for muscle regeneration. Dp71 mRNA was in low abundance (~1% of total dp427m levels – Figure 8D) but was detected in all samples, exhibited a modest but significant decrease with age, and was moreover consistently elevated by approximately 2-fold in DE50-MD muscle.

We next measured markers of dystrophic pathology, including muscle regeneration, myoblast differentiation, fibrotic remodelling and inflammation. For these comparisons, healthy muscle samples with histological evidence of iatrogenic damage were excluded (see Methods), though analysis was also repeated with the entire sample cohort (see extended data, supplementary figure 2 ^43^).

***Embryonic and neonatal myosin heavy chains*.** Skeletal muscle regeneration recapitulates features of muscle development, including expression of embryonic myosin ( *MYH3*) within myotubes during early regeneration, followed by neonatal myosin ( *MYH8*) within the maturing myofibres associated with later regeneration ^40^. Immunolabelling for embryonic myosin heavy chain revealed prominent regenerating fibres within DE50-MD skeletal muscle ( Figure 3): measurement of gene expression allows this phenomenon to be quantified. Expression of both embryonic and neonatal myosin heavy chain mRNAs was prominently elevated in DE50-MD muscle, present at levels 2-3 orders of magnitude greater than in healthy muscle ( Figure 9A, B). This difference remained highly significant at all ages, suggesting that DE50-MD muscle maintained a robust regenerative response throughout the study period. Moreover, while neonatal myosin expression was detected at modest levels in healthy muscle (albeit declining with age), healthy expression of embryonic myosin was largely consistent with transcriptional noise (Cq values of 27–33). This suggests that embryonic myosin is canonically not expressed in healthy post-natal muscle, and consequently represents a potent diagnostic marker of regeneration. Notably, higher expression of both embryonic and neonatal myosin was found within healthy samples excluded on grounds of iatrogenic damage (though differences between groups remained highly significant even after inclusion of these specific samples – see extended data, supplementary figure 2 ^43^).

***MEF2C and Myf5*.** Immunolabelling suggested MEF2 expression in both healthy and dystrophic muscle, but with marked enrichment of MEF2 positive cells within regions of regenerating muscle ( Figure 3), consistent with proliferating/differentiating myoblasts. Furthermore, we have previously demonstrated that *MEF2C* and *Myf5* are expressed at elevated levels in DE50-MD skeletal muscle ^55^. *MEF2C* is a master regulator of muscle expression, essential for skeletal muscle development ^65, 66, 78^ while *Myf5* is a member of the basic helix-loop-helix (bHLH) myogenic transcription factor family, associated with early myoblast proliferation and differentiation ^79^ (including expression of *MEF2C* ^80^). Accordingly, we measured expression of these two markers in our longitudinal sample cohort. Expression of *MEF2C* ( Figure 9C) was robust in all samples (Cq 19-22), supporting the role of this factor in healthy muscle maintenance, but was consistently ~2-fold higher in DE50-MD muscle. As with embryonic and neonatal myosin, *MEF2C* expression was typically also elevated in injured healthy muscle (see extended data, supplementary figure 2 ^43^). Expression of *Myf5* was more modest than *MEF2C*, but also significantly different between genotypes ( Figure 9D), and to a greater extent (~5-fold higher in DE50-MD muscle from 3–12 months of age). *Myf5* expression also showed less association with injured healthy muscle, suggesting that elevated expression of this marker following injury is transient under healthy conditions. *Myf5* thus might represent a strong marker of differentiation associated with ongoing regeneration.

***Collagen 1*.** We next measured expression of collagen 1, a fibrosis/connective tissue marker ^81^, using primers targeted to the α1 subunit ( *COL1A1*, Figure 9E). Over the entire sample cohort, gene expression was significantly higher in DE50-MD muscle, but age-specific differences were also substantial: at the 9-month time point expression was similar between genotypes, suggesting that this gene exhibits complex behaviour (accordingly, analysis indicated a strong interaction effect for this biomarker). Differences in collagen expression were comparatively modest in younger muscle, becoming pronounced only at later time points (12–18 months of age), where expression in healthy muscle declined substantially while dystrophic expression remained high. Such timing implies canonical *COL1A1* expression might be associated with muscle growth, and moreover that expression associated with injury might be highly variable. Consistent with this, *COL1A1* in healthy samples excluded on grounds of injury was indeed variable (see extended data, supplementary figure 2 ^43^).

***Osteopontin*.** To assess muscle inflammation, we measured expression of secretory phosphoprotein 1 (SPP1, also known as osteopontin). Encoding an integrin-binding secretory signalling protein, SPP1 has been shown to be a potent marker for inflammatory and tissue remodelling states, including DMD and animal models of dystrophin deficiency ^82– 84^. As shown in Figure 9F, *SPP1* expression was dramatically elevated in DE50-MD muscle with expression in younger dystrophic muscle (3–9 months) being typically higher than in older muscle, suggesting a peak in inflammatory response associated with muscle growth (as seen with acid phosphatase staining, Figure 7). Of particular interest, unlike markers of regeneration (embryonic and neonatal myosin) expression of *SPP1* in injured healthy muscle was not markedly elevated (see extended data, supplementary figure 2 ^43^), showing that this inflammatory marker allows damage specifically as a consequence of dystrophic pathology to be clearly distinguished from iatrogenic damage of otherwise healthy muscle.

***Utrophin*.** Finally, we measured expression of utrophin ( *UTRN*, Figure 9G), the autosomal paralog of dystrophin. Canonically found at the myotendinous and neuromuscular junctions, utrophin is also found at sites of regeneration ^85^, and exhibits compensatory upregulation in the *mdx* mouse ^86^. Collectively, levels of utrophin expression were significantly higher in DE50-MD muscle, suggesting a degree of compensatory change, however differences were also modest, and rarely significant when segregated by age.

### Longitudinal analysis: power calculations

To determine empirically which of the measurements investigated above would be most useful to pre-clinical therapeutic evaluation, we calculated statistical power using the software package GLIMMPSE ^62^ (see methods). Taking our longitudinal cohort data as a basis (with injured healthy samples omitted where indicated) we determined the sample sizes needed to identify, with a statistical power of 0.8, changes in each biomarker corresponding to 25%, 50%, 75% and 100% restoration toward healthy levels. Most metrics necessitated unrealistic numbers of animals for detection of modest changes (25%), however 50% or greater changes could typically be achieved with more practical group sizes ( Table 2). Correspondingly, we further determined for each biomarker the power (i.e. probability of detecting a difference when one exists) that could be confidently detected using N=6 per group. Our data suggests that several muscle biomarkers are readily capable of identifying disease amelioration at the 50% level: for histology, fibre diameter CoV, AP and PSR staining; for qPCR, *MYH3*, *MYH8*, *Myf5*, *COL1A1* and *SPP1*. The two myosin heavy chain genes in particular demonstrated sufficient statistical power (>0.8) to detect changes of only 25% with a group size of N=6. Inclusion of injured healthy samples fractionally lowered statistical power of gene expression metrics, with the notable exception of *SPP1*, which remained robust to iatrogenic artefact (again supporting the utility of this biomarker -see underlying data ^63^).

**Table 2.** Power calculations for candidate biomarkers in vastus lateralis longitudinal analysis. Leftmost column: biomarkers (Fibre CoV: coefficient of variation in minimum Feret diameter; PSR: picrosirius red fibrosis fraction; AP: acid phosphatase fraction (log10); Dp427 ex62: qPCR of dystrophin exons 62-64; Dp71: qPCR of dystrophin dp71 isoform; *MYH3*: qPCR for embryonic myosin heavy chain; *MYH8*: qPCR for neonatal myosin heavy chain; *MEF2C*: qPCR for myocyte enhancer factor 2C; *Myf5*: qPCR for bHLH factor *Myf5*; *COL1A1*: qPCR for collagen 1; *SPP1*: qPCR for osteopontin; *UTRN*: qPCR for utrophin). Left panel: group sizes need to detect changes in DE50-MD metrics toward WT values, corresponding to 100%, 75%, 50% and 25%, with statistical power >0.8. All comparisons necessitating group sizes >6 are shaded. Right panel: statistical power of each biomarker for assessing 100%, 75%, 50% and 25% changes, given N=6 per group. All comparisons with statistical power <0.8 are shaded. * = comparisons made with injured healthy muscle samples omitted.

	Group size to detect change (%)				Statistical power with N=6			
	100%	75%	50%	25%	100%	75%	50%	25%
Fibre CoV	3	3	5	13	1.000	1.000	0.955	0.452
PSR	3	3	4	12	1.000	1.000	0.964	0.473
AP	2	3	4	9	1.000	1.000	0.993	0.605
Dp427 ex62	4	5	10	36	0.987	0.885	0.562	0.186
Dp71	3	4	8	26	0.999	0.962	0.705	0.241
MYH3 *	2	2	3	6	1.000	1.000	1.000	0.837
MYH8 *	2	2	3	5	1.000	1.000	1.000	0.911
MEF2C *	3	4	7	22	1.000	0.983	0.782	0.280
Myf5 *	3	4	6	20	1.000	0.990	0.819	0.303
COL1A1 *	3	4	6	20	1.000	0.990	0.818	0.302
SPP1 *	2	3	3	8	1.000	1.000	0.998	0.683
*UTRN*	5	8	16	59	0.904	0.690	0.375	0.130

### Body-wide analysis: post-mortem histology

Having assessed multiple quantitative metrics in age-matched healthy and DE50-MD *vastus* muscle to identify those of most (and least) potential utility, we next assessed the more promising markers in a body-wide context, using a broad selection of muscles collected post-mortem (from a subset of the animals used in our longitudinal study). To ensure representative coverage, this panel of muscles spanned the entire body, with nine samples collected from the pelvic limb, two from the thoracic limb, three from the trunk (including the diaphragm and intercostals, critical muscles of respiration) and three masticatory muscles (see methods and Figure 1C). Our post-mortem cohort consisted of three groups: healthy and dystrophic animals euthanised at 18 months (WT N=3; DE50-MD N=4) and a third set of samples taken from DE50-MD animals euthanised at 5–7 months due to dysphagia (N=4). This latter group permitted assessment of more generalised pathological features during the peak (3–9 month) inflammatory period identified above ( *vastus* samples from this group were highly comparable with age-matched non-dysphagic animals, indicating that dysphagia was not reflective of greater systemic disease severity -see extended data, supplementary file 1 ^43^).

**Figure 10.**
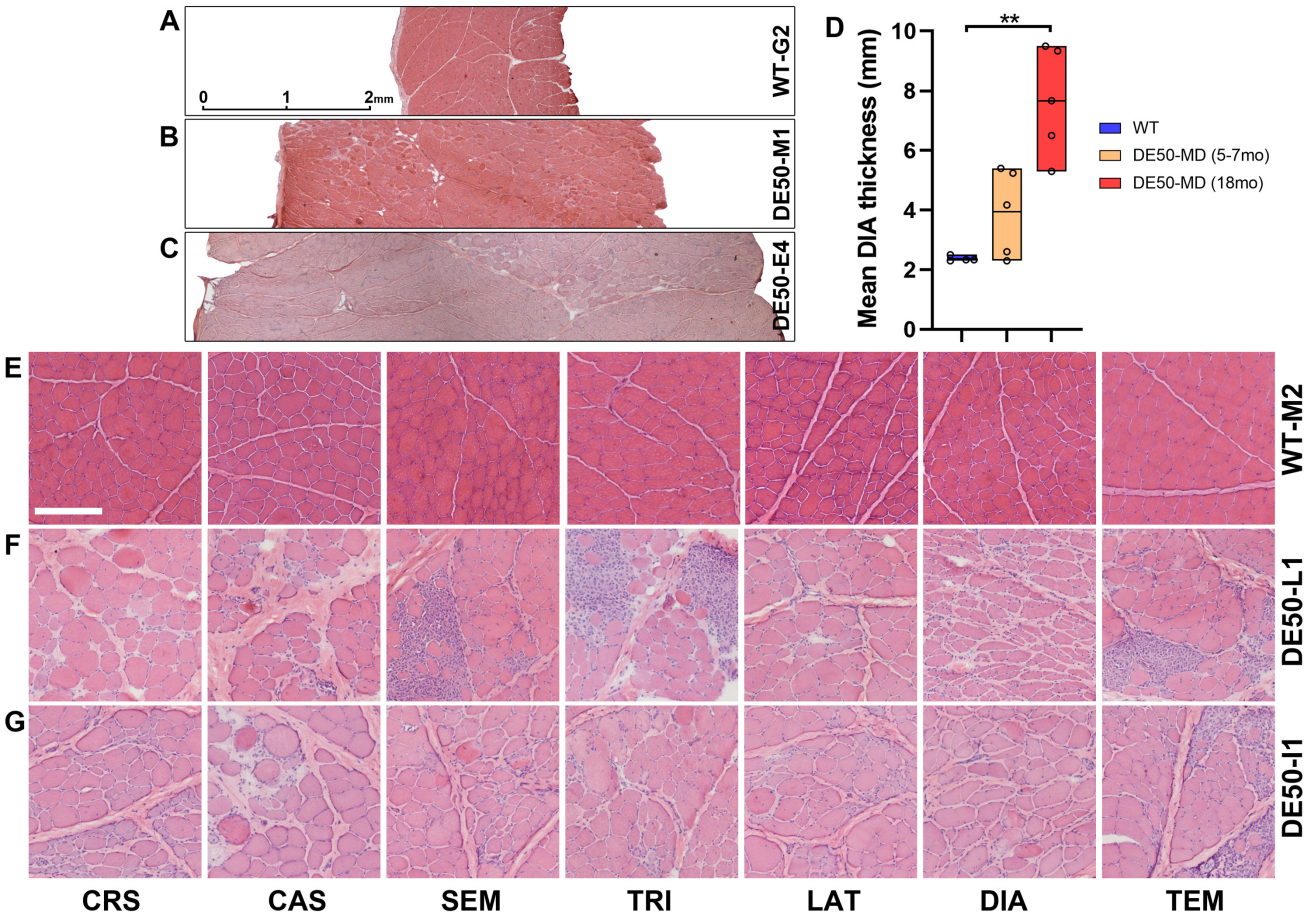
Post-mortem H&E shows dystrophic pathology differs between muscles. Low magnification Haematoxylin and Eosin (H&E) images of histological sections of post-mortem diaphragm samples from ( **A**) 18-month-old WT (WT-G2); ( **B**) 6-month-old DE50-MD (DE50-M1); ( **C**) 18-month-old DE50-MD (DE50-E4) demonstrate hypertrophy of dystrophic diaphragms (scale bar: 2mm). Measured mean diaphragm thickness values ( **D**) suggested pathological hypertrophy increased with age (WT, N=4; DE50-MD 5–7 months, N=5; DE50-MD 18 months, N=5). ( **E**- **G**) Representative higher-power H&E images of histological sections prepared from post-mortem muscle samples of Cranial sartorius (CRS), Caudal sartorius (CAS), Semimembranosus (SEM), Triceps (TRI), Latissimus dorsi (LAT), Diaphragm (DIA) and Temporalis (TEM). Sections shown were collected at 18 months of age from ( **E**) one healthy dog (WT-J1), and DE50-MD dogs at ( **F**) 7 months of age (DE50-L1) and ( **G**) 18 months of age (DE50-I1). WT muscles appeared healthy, while muscles from DE50-MD dogs exhibited dystrophic pathology that varied with muscle group and animal age. Focal nuclear-rich patches of degeneration/regeneration were typically more abundant at 7 months, while fibrotic accumulation was more pronounced at 18 months, but fibrotic changes were marked even at 7 months in CAS and DIA muscles. Scalebar: 200µm.

***Muscle pathology: Haematoxylin and eosin*.** Previous work indicated that DE50-MD dogs exhibit significant muscle wasting: MRI analysis of the pelvic limb and lumbar regions revealed most muscle volumes were typically markedly lower in dystrophic animals ^36^, findings supported by fibre diameter measurements in the *vastus* ( Figure 4). Post-mortem sampling revealed the diaphragm to be a notable exception to this trend ( Figure 10A–C). This muscle exhibited characteristic age-associated pathological hypertrophy in dystrophic animals, thickening by as much as 4-fold at 18 months of age ( Figure 10D). More broadly, H&E staining of muscle sections ( Figure 10E–G) confirmed the widespread nature of dystrophic changes in DE50-MD muscle (with increased fibre size variation and disrupted fascicular architecture), but also highlighted differences both as a function of age and muscle. Focal cell-dense regions of profound degeneration/regeneration were present in all muscles but were more frequently observed at ~6 months of age than at 18 months, again suggesting that DE50-MD dogs experience a peak in degenerative/inflammatory pathology within the first year of life (as shown in longitudinal findings, above). Fibrotic changes, conversely, were more marked in 18-month-old samples, and older muscle typically exhibited widespread endomysial thickening as well as disruption of fascicular architecture (though these changes were also present in ~6-month-old *caudal sartorius* (CAS) and diaphragm (DIA) muscles - Figure 10F).

**Figure 11.**
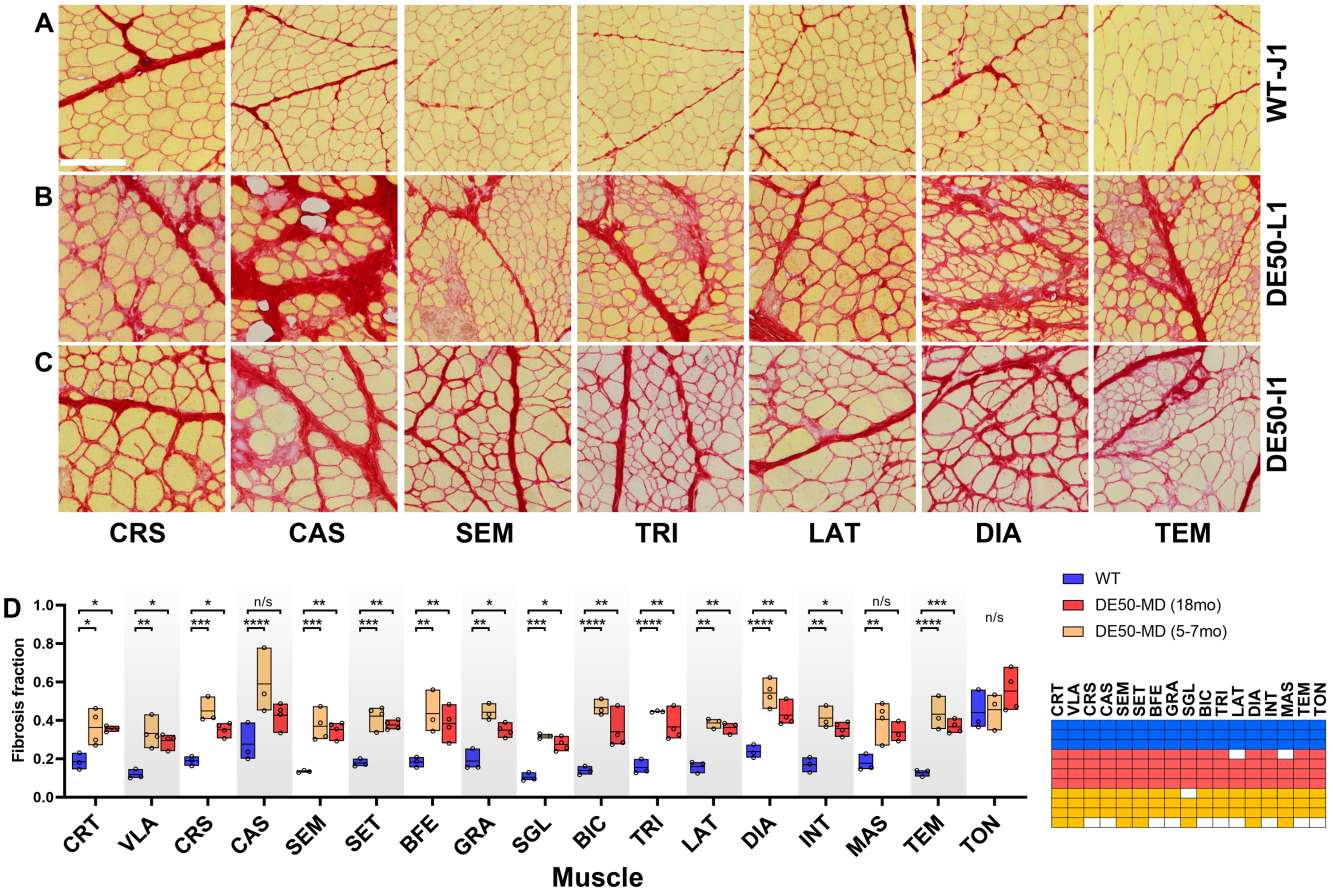
Post-mortem PSR analysis reveals muscle-specific differences in fibrosis. Representative picrosirius red (PSR) images of histological sections prepared from post-mortem muscle samples of Cranial sartorius (CRS), Caudal sartorius (CAS), Semimembranosus (SEM), Triceps (TRI), Latissimus dorsi (LAT), Diaphragm (DIA) and Temporalis (TEM). Sections shown were collected at 18 months of age from ( **A**) one healthy dog (WT-J1), and DE50-MD dogs at ( **B**) 7 months of age (DE50-L1) and ( **C**) 18 months of age (DE50-I1). Levels of healthy connective tissue varied between muscles but remained modest, while dystrophic muscle exhibited prominent increases in both perimysial and endomysial content. All underlying images, including muscles not shown (Cranial Tibial (CRT); Vastus lateralis (VLA); Semitendinosus (SET); Biceps femoris (BFE); Gracilis (GRA); Superficial gluteal (SGL); Biceps (BIC); Intercostal (INT); Masseter (MAS); Tongue (TON)) can be found in the underlying data. Analysis of mean per-muscle fibrosis fractions for the entire post-mortem cohort ( **D**) revealed a significant effect of group (P<0.001) and muscle type (P<0.0001), but also significant interaction (P<0.05) indicating muscle-specific variability. Post-hoc multiple comparisons within muscles are shown (Linear mixed model, Holm-Šídák correction: * = P<0.05; ** = P<0.01; *** = P<0.001; **** = P<0.0001; n/s = not significant). No significant differences between young and old DE50-MD groups were detected for any muscle. Boxes indicate means and range, open circles denote measured individual values. 51 samples from 3 WT animals, 66 samples from 4 (18-month-old) DE50-MD animals, and 57 samples from 4 DE50-MD (5–7-month-old) animals were used for these data to give N values of 3–4 per muscle, per group (see sample key, lower right). Scalebar: 200µm.

***Muscle fibrosis: Picrosirius red*.** Post-mortem samples stained with PSR ( Figure 11A–C) revealed widespread muscle fibrosis in DE50-MD dogs, with almost all muscles showing clear increases in connective tissue content both at the endomysial and perimysial level. The *caudal sartorius* (CAS) and diaphragm (DIA) were particularly severely affected (as suggested via H&E, Figure 10), and PSR staining also revealed profound hypertrophy-associated fibre splitting: principally found in the diaphragm, where this was evident even at 6 months. Quantitative analysis ( Figure 11D) revealed modest muscle-specific differences even between healthy samples (presumably due to differences in fibre type compositions, and hence fibre diameters), but individual values for any given muscle were typically comparable within a group, especially at 18 months. Fibrosis fractions for both young and old DE50-MD samples were significantly higher than those of healthy samples for most muscles, with *biceps* (BIC), *triceps* (TRI), diaphragm and *temporalis* showing the most consistent differences. Measured fibrosis fractions were typically higher at ~6 months than at 18 months in DE50-MD samples, but as with the *vastus* ( Figure 6), this likely reflects smaller median fibre diameter -and hence surface area to perimeter ratio- in younger samples, rather than greater fibrosis *per se*. Intriguingly, no significant differences in fibrosis fraction were found for the tongue (TON) muscle, at any age.

**Figure 12.**
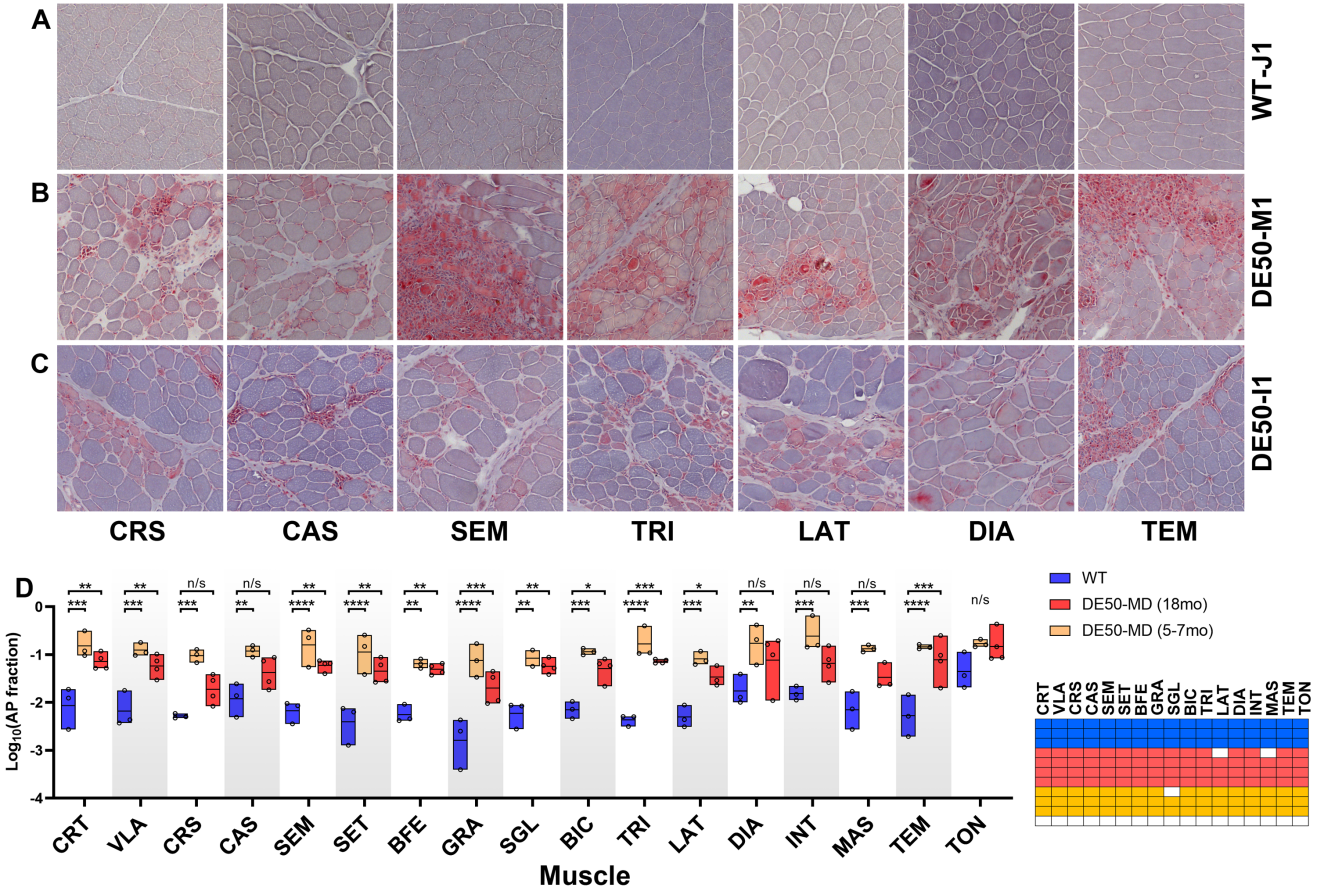
Post-mortem acid-phosphatase analysis reveals age and muscle-specific differences in inflammation. Representative acid phosphatase (AP) images of histological sections prepared from post-mortem muscle samples of Cranial sartorius (CRS), Caudal sartorius (CAS), Semimembranosus (SEM), Triceps (TRI), Latissimus dorsi (LAT), Diaphragm (DIA) and Temporalis (TEM). Sections shown were collected at 18 months of age from ( **A**) one healthy dog (WT-J1), and DE50-MD dogs at ( **B**) 7 months of age (DE50-M1) and ( **C**) 18 months of age (DE50-I1). Dystrophic muscle exhibited profound increases in acid-phosphatase activity, most prominently at younger ages. All underlying images, including muscles not shown (Cranial Tibial (CRT); Vastus lateralis (VLA); Semitendinosus (SET); Biceps femoris (BFE); Gracilis (GRA); Superficial gluteal (SGL); Biceps (BIC); Intercostal (INT); Masseter (MAS); Tongue (TON)) can be found in the underlying data. Analysis of log transformed per-muscle AP fractions for the entire post-mortem cohort ( **D**) revealed significant differences between groups (P<0.001) and between muscles (P<0.0001). Post-hoc multiple comparisons within muscles are shown (Linear mixed model, Holm-Šídák correction: * = P<0.05; ** = P<0.01; *** = P<0.001; **** = P<0.0001; n/s = not significant). No significant differences between young and old DE50-MD groups were detected for any muscle. Boxes indicate means and range, open circles denote measured individual values. 51 samples from 3 WT animals, 66 samples from 4 (18-month-old) DE50-MD animals, and 50 samples from 3 DE50-MD (5–7-month-old) animals were used for these data to give N values of 3–4 per muscle, per group, with the exception of superficial gluteal (SGL, N=2 for young DE50-MD: see sample key, lower right). Scalebar: 200µm.

***Muscle inflammation: Acid phosphatase*.** AP staining of post-mortem samples supported our longitudinal findings: while healthy muscle tissue was predominantly consistent with counterstain only ( Figure 12A), younger dystrophic muscle exhibited prominent focal regions of cell-dense acid phosphatase activity (consistent with inflammatory infiltrates and degenerating myofibres - Figure 12B), while older dystrophic muscle was typically host to more diffuse intra-fibre staining ( Figure 12C). Image quantification confirmed these observations: measured log(AP fraction) values were typically higher in all DE50-MD samples, but elevated to a greater and more consistent extent in younger tissue ( Figure 12D) -an observation that cannot be attributed to fibre diameter (unlike PSR), and that again supports a peak inflammatory phase within the first year of life. Post-hoc multiple comparisons indicated significant differences between healthy and dystrophic samples for the majority of muscles (particularly between healthy and young DE50-MD, reflecting the more extensive staining in younger dystrophic muscle), and the *gracilis* (GRA), *triceps* (TRI) and *temporalis* (TEM) exhibited the most consistent differences regardless of age. Several muscles were not significantly different between 18-month-old healthy and DE50-MD dogs, including both respiratory muscles (diaphragm and intercostal), suggesting that the inflammatory peak (and subsequent decline) might occur earlier in these critical muscles. Similar to PSR staining, the tongue uniquely was comparable between all three groups, indicating that this muscle might be of limited diagnostic value.

### Body-wide analysis: post-mortem gene expression

As with our longitudinal study of the *vastus*, we next measured gene expression via qPCR, using RNA isolated from serial sections of the same samples used for histological analyses, above. Given the limited statistical value of *MEF2C* and *UTRN* (see Figure 9), these markers were not measured.

**Figure 13.**
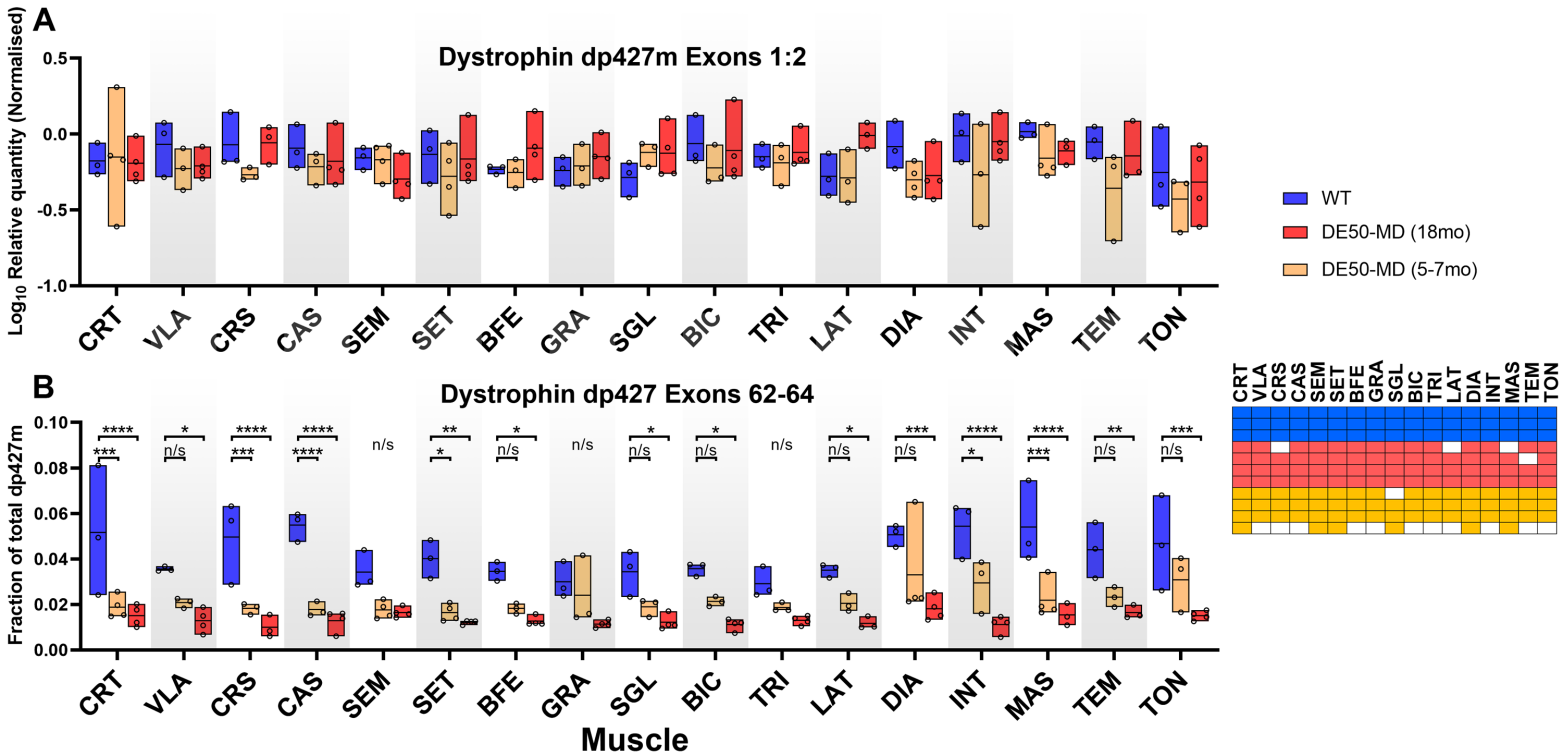
Post-mortem analysis of dystrophin expression. Quantitative PCR (qPCR) analysis of per-muscle dystrophin expression in the entire post-mortem sample cohort (Cranial Tibial (CRT); Vastus lateralis (VLA); Cranial sartorius (CRS); Caudal sartorius (CAS); Semimembranosus (SEM); Semitendinosus (SET); Biceps femoris (BFE); Gracilis (GRA); Superficial gluteal (SGL); Biceps (BIC); Triceps (TRI); Latissimus dorsi (LAT); Diaphragm (DIA); Intercostal (INT); Masseter (MAS); Temporalis (TEM); Tongue (TON)) using primers targeting dp427m exons 1–2 to measure total transcription ( **A**) and dystrophin exons 62–64 to measure more mature transcripts ( **B**) (see Figure 8E for schematic). Levels of dystrophin transcriptional initiation (exons 1–2) were comparable between groups, with only mild muscle-specific variation (P<0.05, muscle), while levels of mature transcripts (exons 62–64) were markedly reduced in both young and old DE50-MD muscle, reflecting transcript degradation via nonsense mediated decay (NMD) (P<0.001, group; P<0.0001, muscle; P<0.05, interaction). Post-hoc multiple comparisons within muscles are shown (Linear mixed model, Holm-Šídák correction: * = P<0.05; ** = P<0.01; *** = P<0.001; **** = P<0.0001; n/s = not significant). No significant differences between young and old DE50-MD groups were detected for any muscle. Boxes indicate means and range, open circles denote measured individual values. Exon 1–2 data is shown as log10 of relative quantity (RQ) values (normalised to the geometric mean of *SDHA*, *RPL13a* and *HPRT1*), while Exon 62–64 data is normalised to total dystrophin (Dp427 exon 1–2). 51 samples from 3 WT animals, 64 samples from 4 (18-month-old) DE50-MD animals, and 56 samples from 3 DE50-MD (5–7-month-old) animals were used for these data to give N values of 3–4 per muscle, per group (see sample key, right).

***Dystrophin*.** Using primers to dp427m exon 1-2 (nascent dystrophin mRNA) and dp427 exon 62-64 (predominantly mature transcripts -see Figure 8E) we measured dystrophin expression in our post-mortem samples. As with *vastus*, levels of nascent dp427m ( Figure 13A) were not significantly different between genotypes, and showed only modest differences between muscles. In contrast, exon 62-64 sequence ( Figure 13B) was strikingly different between groups, with markedly lower levels in dystrophic samples, consistent with nonsense-mediated clearance of DE50-MD transcripts. Similar to *vastus* data, however, measured expression levels also varied considerably between individual samples within groups, and consequently not all muscles exhibited statistical differences following post-hoc multiple comparisons analysis.

**Figure 14.**
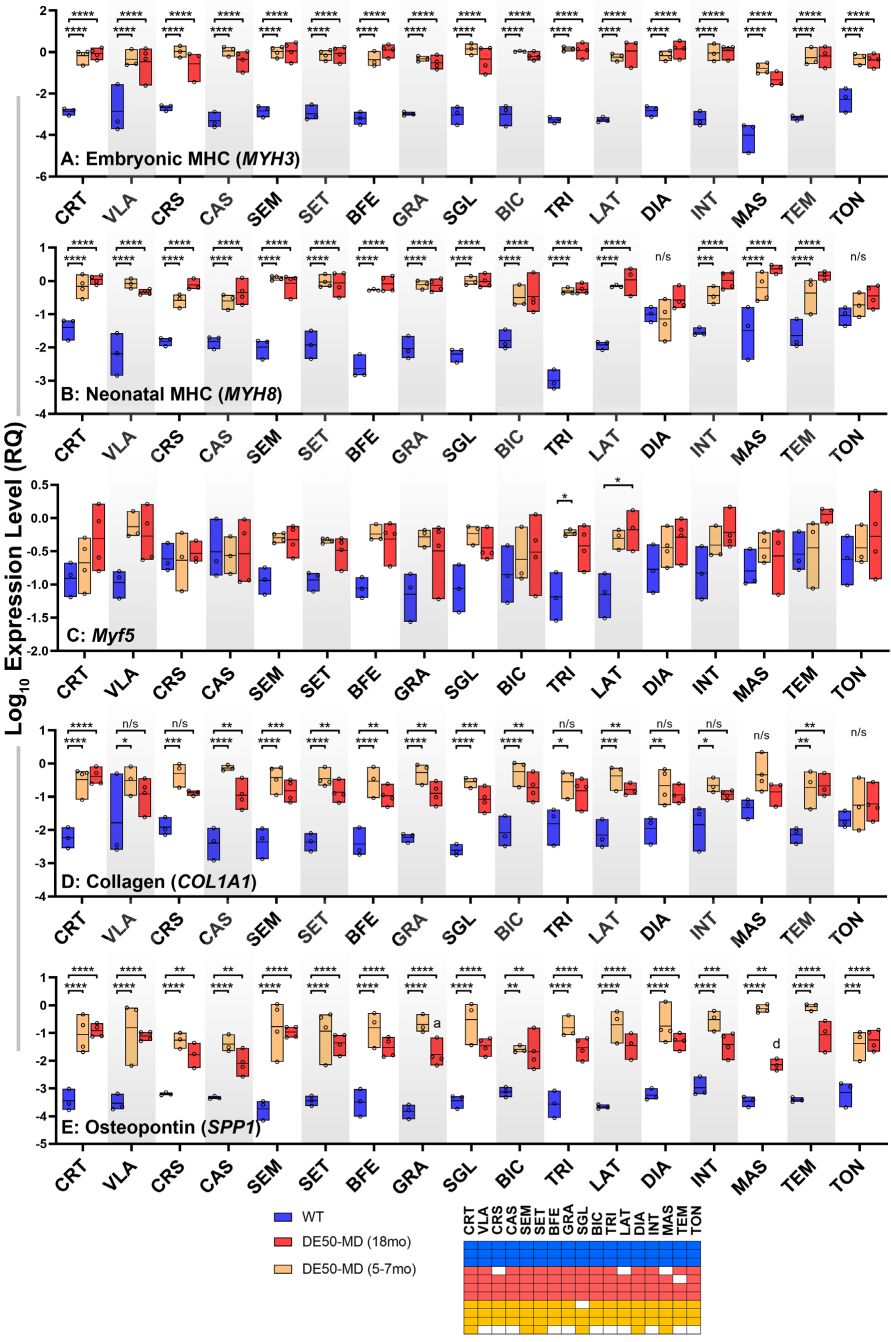
Post-mortem gene expression: markers of regeneration, inflammation and fibrosis. Quantitative PCR (qPCR) analysis of per-muscle gene expression in the entire post-mortem sample cohort (Cranial Tibial (CRT); Vastus lateralis (VLA); Cranial sartorius (CRS); Caudal sartorius (CAS); Semimembranosus (SEM); Semitendinosus (SET); Biceps femoris (BFE); Gracilis (GRA); Superficial gluteal (SGL); Biceps (BIC); Triceps (TRI); Latissimus dorsi (LAT); Diaphragm (DIA); Intercostal (INT); Masseter (MAS); Temporalis (TEM); Tongue (TON)) using primers to ( **A**) *MYH3* (embryonic myosin) and ( **B**) *MYH8* (neonatal myosin). Both regenerative markers were highly expressed in all DE50-MD muscles, in most cases orders of magnitude greater than in healthy tissue, particularly for *MYH3* (P<0.0001, group; P<0.0001, muscle; P<0.05, interaction). *MYH8* exhibited strong muscle-specific behaviour (P<0.0001, group; P<0.0001, muscle; P<0.0001, interaction), being expressed at comparable levels specifically in healthy DIA and TON. Primers to *Myf5* ( **C**) revealed higher expression in DE50-MD muscle overall (P<0.05, group; P<0.01, interaction), but this was not significant within most individual muscles. Expression of *COL1A1* (collagen 1) ( **D**) similarly indicated global increases in dystrophic, but did not reach significance within every muscle measured, especially masticatory muscles muscle (P<0.001, group; P<0.01, interaction). Expression of *SPP1* (osteopontin) ( **E**) was significantly elevated in all DE50-MD muscles (P<0.0001, group; P<0.01, interaction) and expression in younger DE50-MD samples was typically higher than that in older DE50-MD muscle. Post-hoc multiple comparisons within muscles are shown for each marker (Linear mixed model, Holm-Šídák correction: * = P<0.05; ** = P<0.01; *** = P<0.001; **** = P<0.0001; n/s = not significant). No significant differences between young and old DE50-MD groups were detected for any marker except *SPP1* (a = P<0.05; b = P<0.01; c = P<0.001; d = P<0.0001). Boxes indicate means and range, open circles denote measured individual values. All data is shown as log10 of relative quantity (RQ) values (normalised to the geometric mean of *SDHA*, *RPL13a* and *HPRT1*). 51 samples from 3 WT animals, 64 samples from 4 (18-month-old) DE50-MD animals, and 56 samples from 3 DE50-MD (5–7-month-old) animals were used for these data to give N values of 3–4 per muscle, per group (see sample key, bottom).

***Embryonic and neonatal myosin heavy chains*.** Expression of embryonic ( *MYH3*) and neonatal ( *MYH8*) myosin heavy chains was highly predictive in our longitudinal data ( Figure 9A, B). Measurement of *MYH3* in post-mortem samples ( Figure 14A) confirmed the utility of this biomarker: expression in healthy samples was again largely consistent with stochastic transcriptional noise (Cq>30) but was robustly elevated by 2–3 orders of magnitude in all DE50-MD muscles, regardless of age (Cq ~20–24) and was highly significant for every muscle examined (including the tongue). Expression of *MYH8* was conversely more nuanced ( Figure 14B): while expression was high in all dystrophic samples (Cq ~17–20), modest levels of this transcript were also found in healthy muscle (Cq ~24–26, as shown in the *vastus*, Figure 9B). Disease-associated increases were nevertheless highly significant for the majority of muscles, with the exception of the diaphragm and tongue specifically, where levels of *MYH8* expression were comparable between healthy and dystrophic samples. Our data here suggests that even in healthy animals these specific muscles canonically express this myosin heavy chain at comparatively high levels.

***Myf5*.** Expression of the myogenic transcription factor *Myf5* represented a strong candidate biomarker in longitudinal vastus analysis ( Figure 9D), however this marker proved to be unexpectedly poor when examined in a body-wide context ( Figure 14C): while elevated in dystrophic muscle overall, increases were often modest and rarely reached significance at the individual muscle level. For some muscles, such as the *cranial* and *caudal sartorius* (CRS and CAS), expression was entirely comparable between genotypes, suggesting substantial muscle-specific behaviour for this marker.

***Collagen 1*.** *COL1A1* expression was more informative: levels in young and old DE50-MD samples were comparable, but both were clearly elevated over healthy tissue for most muscles. Correspondingly, many per-muscle comparisons were significant, being particularly robust in the *cranial tibial* (CRT), *semimembranosus* (SEM) and *superficial gluteal* (SGL) muscles. A degree of muscle-specific behaviour was however present, with the tongue again showing no significant difference between groups, here also accompanied by the *masseter* (MAS).

***Osteopontin*.** *SPP1* (osteopontin) was a strong candidate in *vastus* muscle, with this inflammatory marker uniquely able to discern muscle undergoing dystrophic damage from muscle damaged iatrogenically. *SPP1* remained a strong marker under post-mortem analysis, being present at markedly elevated levels in all DE50-MD muscles studied. In agreement with a peak inflammatory phase during the first year of life, expression in young DE50-MD muscle was often higher than in older samples -significantly so in the *gracilis* (GRA) and *masseter* (MAS) muscles- but our data suggests the inflammatory response is still prominent and widespread at 18 months (including muscles that performed poorly via acid phosphatase, such as the diaphragm). Expression of *SPP1* even revealed highly significant differences between healthy and dystrophic tongue muscles.

### Post-mortem analysis: power calculations

As with our longitudinal analysis, the primary goal of these investigations was to determine the statistical utility of these skeletal muscle biomarkers for future work. The body-wide assessment offered by post-mortem analysis is a potentially powerful repeated measures approach, and consequently the repeated measures modelling used with the *vastus* data (same muscle, multiple time points) can readily be applied to post-mortem data (multiple muscles, same time point), permitting equivalent power calculations. The GLIMMPSE package allows for a maximum of 10 repeated measures ^62^, thus we selected a subset of muscles from our panel of 17, favouring those exhibiting strongly significant differences between genotypes via multiple biomarkers, but also weighting for clinical relevance, histological tractability, and ensuring at least one representative muscle from pelvic limb, thoracic limb, trunk and masticatory categories (CRT, VLA, CRS, SEM, BFE, BIC, TRI, LAT, DIA, MAS). Comparisons were made in pairwise fashion (WT vs DE50-MD 18 month, WT vs DE50-MD 5–7 month, and for consistency, 5–7 month DE50-MD vs 18 month DE50-MD) and as before for each metric we assessed the group sizes needed to detect (with a statistical power >0.8) ameliorative changes corresponding to 25%, 50%, 75% and 100% restoration to healthy levels, and the statistical power achievable at each threshold given a proposed group size of N=6. As expected, young and old DE50-MD samples were highly comparable, with most metrics demanding group sizes of hundreds or thousands of animals necessary to detect modest differences (see underlying data ^63^). Comparison of 18-month-old healthy data with DE50-MD muscle at 5–7 months ( Table 3) or 18 months ( Table 4) in contrast demonstrates the statistical advantages offered by post-mortem analysis: for almost all biomarkers assessed, a group size of 6 animals is sufficient to detect even a 25% improvement toward healthy values (one notable exception being *Myf5* gene expression, which performed poorly in a post-mortem context – see Figure 14C), and for many markers our data suggest such changes could be detected with yet smaller group sizes (or that N=6 is sufficient to detect yet smaller changes).

**Table 3.** Power calculations for candidate biomarkers in post-mortem analysis: WT (18 months) vs DE50-MD (5–7 months). Leftmost column: biomarkers (PSR: picrosirius red fibrosis fraction; AP: acid phosphatase fraction (log10); Dp427 ex62: qPCR of dystrophin exons 62–64; *MYH3*: qPCR for embryonic myosin heavy chain; *MYH8*: qPCR for neonatal myosin heavy chain; *Myf5*: qPCR for bHLH factor *Myf5*; *COL1A1*: qPCR for collagen 1; *SPP1*: qPCR for osteopontin). Left panel: group sizes need to detect changes in DE50-MD metrics toward WT values, corresponding to 100%, 75%, 50% and 25%, with statistical power >0.8, using post-mortem data from CRT, VLA, CRS, SEM, BFE, BIC, TRI, LAT, DIA and MAS muscles (see Figure 1). Comparisons necessitating group sizes >6 are shaded. Right panel: statistical power of each biomarker for assessing 100%, 75%, 50% and 25% changes, given N=6 per group. All comparisons with statistical power <0.8 are shaded.

	Group size to detect change (%)				Statistical power with N=6			
	100%	75%	50%	25%	100%	75%	50%	25%
PSR	2	2	2	4	1.000	1.000	1.000	0.997
AP	2	2	2	3	1.000	1.000	1.000	0.998
Dp427 ex62	2	3	3	8	1.000	1.000	0.997	0.670
*MYH3*	2	2	2	3	1.000	1.000	1.000	1.000
*MYH8*	2	2	2	3	1.000	1.000	1.000	0.998
*Myf5*	3	3	5	13	1.000	1.000	0.950	0.442
*COL1A1*	2	2	3	6	1.000	1.000	1.000	0.850
*SPP1*	2	2	2	3	1.000	1.000	1.000	1.000

**Table 4.** Power calculations for candidate biomarkers in post-mortem analysis: WT (18 months) vs DE50-MD (18 months) Leftmost column: biomarkers (PSR: picrosirius red fibrosis fraction; AP: acid phosphatase fraction (log10); Dp427 ex62: qPCR of dystrophin exons 62–64; *MYH3*: qPCR for embryonic myosin heavy chain; *MYH8*: qPCR for neonatal myosin heavy chain; *Myf5*: qPCR for bHLH factor *Myf5*; *COL1A1*: qPCR for collagen 1; *SPP1*: qPCR for osteopontin). Left panel: group sizes need to detect changes in DE50-MD metrics toward WT values, corresponding to 100%, 75%, 50% and 25%, with statistical power >0.8, using post-mortem data from CRT, VLA, CRS, SEM, BFE, BIC, TRI, LAT, DIA and MAS muscles (see Figure 1). Comparisons necessitating group sizes >6 are shaded. Right panel: statistical power of each biomarker for assessing 100%, 75%, 50% and 25% changes, given N=6 per group. All comparisons with statistical power <0.8 are shaded.

	Group size to detect change (%)				Statistical power with N=6			
	100%	75%	50%	25%	100%	75%	50%	25%
PSR	2	2	3	4	1.000	1.000	1.000	0.984
AP	2	2	3	5	1.000	1.000	1.000	0.889
Dp427 ex62	2	2	3	5	1.000	1.000	1.000	0.956
*MYH3*	2	2	2	3	1.000	1.000	1.000	1.000
*MYH8*	2	2	2	3	1.000	1.000	1.000	0.999
*Myf5*	3	4	6	17	1.000	0.996	0.877	0.348
*COL1A1*	2	3	3	7	1.000	1.000	0.999	0.752
*SPP1*	2	2	2	3	1.000	1.000	1.000	1.000

## Discussion

Canine models of DMD play a key role in the translation of therapies from laboratory to clinic, exhibiting disease severity that closely matches that of human patients, including pathological aspects that are mild or absent in mouse models ^25^, such as widespread muscle atrophy and fibrosis ^24, 26^. The DE50-MD dog model offers further pre-clinical advantages for some therapeutic approaches, carrying a ‘human-like’ mutation (in the hotspot between exons 45 and 54) that can be readily corrected via exon skipping-based therapeutic approaches ^28^. Recognising both the value of this new animal model and the ethical concerns in pre-clinical use of companion animals, we have conducted an extensive natural history trial to characterise the DE50-MD disease phenotype (Wellcome Trust grant 101550). Findings from the work shown here, and findings published elsewhere ^35– 37^, establish a comprehensive picture of disease progression, identifying parameters that robustly distinguish dystrophic animals from their healthy littermates (and those that do not), and further establishing the magnitude and variability of these parameters.

The work described here assessed disease progression within skeletal muscle samples: DMD is principally a muscle disorder, and muscle sampling is consequently an essential component of disease assessment. Muscle biopsy carries several practical concerns, however. In rodents, muscle analysis is a terminal procedure: the small size of these models precludes repeat muscle biopsy necessary for longitudinal assessment. Dogs (like human patients) permit repeat muscle sampling, however biopsy collection remains invasive, necessitates anaesthesia, and unavoidably introduces iatrogenic damage to a tissue already subject to continual cycles of damage and repair. As such, muscle sampling should ideally be performed sparingly, with effort taken to maximise per-sample informational gain ^38^. A broad panel of disease-associated metrics can be collected at the histological level (conventional histochemical stains, IHC for specific protein markers) and at the level of gene expression (qPCR), but tissue samples are precious: to avoid wasting material it is therefore important to determine which measurements are of most statistical value. Metrics can be both qualitative and quantitative, and can address different aspects of dystrophic pathology, not all of which will necessarily respond to therapeutic intervention in a similar fashion. Some metrics might also exhibit substantial variability, either inherently or due to factors beyond disease (such as choice of sampling site or animal age): metrics exhibiting modest but highly consistent disease-associated differences might consequently be favoured over those with more dramatic but variable behaviour. For use in therapeutic trials, especially those using large animal models, biomarkers should ideally be of sufficient statistical power to permit rigorous analysis with comparatively small group sizes.

### Fibre profiles

Muscle fibre profiles exhibit distinctive changes in response to dystrophin deficiency: compensatory hypertrophy leads to increases in minimum Feret diameter (MFD), while fibre regeneration (and splitting of heavily hypertrophic fibres) results in decreases in diameter and increases in (apparent) fibre number. In humans, these processes are largely sequential: hypertrophic changes predominate prior to 5 years of age, with splitting and regeneration overtaking subsequently ^67^. In mice, robust regeneration and compensatory hypertrophy continue into adulthood, resulting in a lower median MFD, but also a broadening of fibre diameter distribution (increased coefficient of variation) ^87^. Both median MFD and CoV thus represent potential quantitative histological metrics. Measurement of fibre profiles in our *vastus* samples ( Figure 4) revealed characteristic shifts in distribution: median MFD values were significantly lower in DE50-MD muscle at all ages except 3 months (where all fibres are markedly smaller, likely masking dystrophy-associated reductions). DE50-MD distributions were also consistently broader, indicating both smaller regenerating fibres and fibre hypertrophy, and consequently CoV values were higher in DE50-MD muscle, even at the 3-month time point, making CoV a comparatively strong biomarker for dystrophic pathology (group sizes of N=6 would detect a 50% shift toward WT values with a power of 0.955 – Table 2. For median MFD the conclusions are more nuanced: the association with animal size prompted us to normalise these data to bodyweights ( Figure 4E), which unexpectedly eliminated essentially all variation in median MFD, ostensibly rendering this metric useless. Indeed, one interpretation of these findings is that carefully measuring thousands of individual fibre diameters ultimately amounts to little more than a laborious and indirect means of estimating the weight of a dog. However, this data warrants slightly deeper consideration: while DE50-MD dogs are indeed typically of lower bodyweight than healthy littermates, this is not reflected in size, *per se*. Analysis of skeletal muscle MRI data (another arm of the same natural history study ^36^) showed that DE50-MD skeletal muscle volumes are typically only 50% of healthy levels, while femur lengths (i.e. overall animal size) are comparable. Supporting this, normalising our median MFD data to femur length largely retained the difference between genotypes ( Figure 4F). Interestingly, by ~12 months, when animal growth was largely complete, median MFD for DE50-MD muscle largely stabilised at a value 25% lower than healthy (~30µm vs ~40µm - Figure 4D). Approximating myofibres to cylinders with consistent length between genotypes, a change in diameter from ~40µm (WT) to ~30µm (DE50-MD) results in a ~45% reduction in volume: close to the value determined via MRI. An implication of our findings here is that not only can reduction in bodyweight be near-exclusively ascribed to lower muscle mass rather than smaller animal size (as expected), but that this reduction in muscle mass is potentially secondary. DE50-MD dogs might not have smaller fibres as a consequence of reduced muscle mass, but instead have reduced muscle mass because of these smaller fibres: reductions in volume appear to represent changes at the individual fibre level, an *en bloc* shift toward smaller fibre diameters, rather than gross fibre loss.

The presence of these smaller fibres at different stages of regeneration, alongside larger fibres undergoing compensatory hypertrophy (which we also observe), would be expected to generate a spectrum of fibre diameters, broadening the size distribution: this we robustly detect. This does not adequately explain why the peak of this distribution (i.e. the most prevalent fibre diameter) should ultimately stabilise at a specific, consistent lower value. Atrophy as a consequence of impaired regenerative innervation might explain some reduction in fibre diameter ^88^, however again it is difficult to explain how this would elicit such a consistent shift in peak MFD. Impaired muscle growth might also contribute, as this requires both hypertrophy and the fusion and addition of further myonuclei to the growing myofiber ^89, 90^: specific defects in these processes could limit attainable fibre size. A further intriguing possibility is that these findings reflect a more fundamental, mechanical implication: that dystrophin deficiency becomes highly deleterious above a specific diameter threshold, and that this shifted peak represents the greatest fibre diameter that is sustainable in DE50-MD muscle. Notably, MFD distributions at 3 months of age (when median MFD is 20–25um) show much smaller differences between genotypes than at 6 or 9 months (when median MFD increases to 30–40um in WT muscle). A corollary of this is that one might expect to see associations with fibre type, as larger type IIX fibres would presumably be more vulnerable than smaller type IIA or type I. Further investigations will be needed to explore this possibility.

Overall, the utility of fibre diameter measurements is offset by the caveats discussed above, and by the time-consuming nature of the analysis: therapeutic amelioration of pathology might be expected to alter these metrics, but measurement might be better employed post-hoc on a case-by-case basis, rather than used as a primary assessment of therapeutic efficacy (accordingly, we did not repeat this analysis for post-mortem samples).

Alongside analysis of fibre profiles, we evaluated numbers of dystrophin positive ‘revertant’ fibres ( Figure 5). The underlying mechanism that generates and maintains revertant fibres is not completely understood: frequency of these dystrophin-positive fibres varies in a mutation-specific manner (and thus differs between animal models ^91^), and transcriptomic/proteomic analysis shows revertant dystrophin results from endogenous skipping of one or more exons ^68, 92, 93^, implying that some mutations more readily lend themselves to spontaneous correction than others. In *mdx* mice (where omission of exon 23 restores dystrophin) revertant fibres are readily detected, and numbers of these fibres also increase with age, expanding in clonal fashion to produce revertant ‘patches’ ^94^. The DE50-MD mutation similarly requires only a single exon to be omitted to restore dystrophin (exon 51), but while revertant fibre were detected (and appeared to increase with age, with concomitant revertant patches), overall numbers were substantially lower than those found in the *mdx* model ^95^, and well below the levels expected following beneficial therapeutic restoration. Our study period included significant age- and disease-associated fibre hypertrophy, necessitating revertant numbers to be expressed as a fraction of total fibres, rather than ‘per unit area’ values more common in equivalent mouse studies: given the time-consuming nature of such detailed analysis, we did not repeat this work in our broader post-mortem sample collection (though anecdotally we do not observe marked differences in revertant numbers between muscles – similar to other models).

### Muscle fibrosis and inflammation

We used several histological stains here: H&E for general histopathology, PSR for fibrosis, and AP for inflammation-associated lysosomal activity. Histological staining of tissue sections offers several advantages over immunohistochemistry: it does not require antibodies (few of which are validated for use in canine tissue); it can be conducted comparatively rapidly (and in large batches); and staining is essentially permanent – unlike immunofluorescence where images must be collected promptly, slides can be archived for future analysis. Qualitative histology can be used to corroborate or clarify data obtained from other approaches (as we show with iatrogenic artefact of healthy muscle), but staining can also offer quantitative potential: our data suggests that both PSR and AP represent useful quantitative stains for fibrotic and inflammatory components (respectively) of dystrophic pathology. Combining these two histological approaches allows a more comprehensive picture of dystrophic pathology to be formed.

Fibrosis is a prominent clinical feature of human DMD: ongoing muscle damage and associated inflammation dysregulates the canonical repair process, leading to progressive deposition of fibrotic scar tissue ^96– 98^. This disrupts muscle architecture, impairing ongoing regeneration and potentially exhausting capacity for repair. Extensive intramuscular fibrosis can also impede delivery of therapeutic agents (such as antisense oligonucleotides) to muscle fibres ^99^. Fibrosis in dystrophic mice, however, is not consistent between muscles: the diaphragm is profoundly affected, but other muscles show minimal fibrotic changes ^23^. The mechanism by which mice escape more generalised fibrosis is not well understood, but this phenomenon limits the utility of mouse models for assessing therapeutic amelioration of muscle fibrosis, or for identifying any fibrotic restrictions in therapeutic efficacy. Animal models that better reflect this aspect of human disease (such as dogs) are consequently valuable tools, especially if muscle fibrosis can be reliably quantified (as with our image analysis approach). Fibrosis fractions in our dystrophic *vastus* samples were significantly higher than equivalent healthy samples at all ages ( Figure 6C), confirming that muscle fibrosis is a feature of DE50-MD pathology, and suggesting that quantitative assessment represents a statistically powerful, age-independent approach. We cannot confidently claim this muscle fibrosis is cumulative, however: indeed, in both groups the mean fibrosis fraction demonstrably decreased with age. Given the non-linear relationship between fibre perimeter and fibre area, this counterintuitive finding most likely reflects growth-associated hypertrophy: normalising to median MFD partially addresses this decline ( Figure 6D). Conclusively identifying progressive *vastus* fibrosis might require older samples: our 3–18-month study period includes the bulk of post-natal animal growth, while equivalent studies showing cumulative diaphragm fibrosis in the mouse typically do not begin until 6–8 weeks of age ^100– 102^, near the end of the peak murine growth phase. Body-wide post-mortem analysis provides further insights, however, as our PSR data demonstrates that the extent of pathological muscle fibrosis varies in a muscle-specific manner ( Figure 11). Some muscles, such as the *temporalis* and diaphragm, show profound increases in connective tissue (and show these increases earlier), while for others, such as the *superficial gluteal* and *latissimus dorsi*, increases appear milder and more gradual. These differences are likely to principally reflect frequency of use (muscles necessary for eating and breathing are employed more regularly than those required for running), supporting a progressive element to muscle fibrosis in this animal model. In the diaphragm there is also evidence of significant fibre splitting, a pathological response to hypertrophy (which is profound in the DE50-MD diaphragm, see Figure 10), and one which substantially increases connective tissue content. Fibrosis in the diaphragm might therefore uniquely reflect an ongoing process of hypertrophy and splitting, occurring alongside the damage-associated fibrotic remodelling found in other muscles.

For the purposes of this study (and pre-clinical trial design) the conclusions are clear: provided analysis is conducted in an age-matched fashion, image quantification of PSR staining represents a strong candidate biomarker. Post-mortem analysis is particularly powerful in this respect, capable of detecting improvements at the 25% level with as few as 4 animals per group.

Alongside PSR, we also investigated acid phosphatase (AP) staining. This stain detects activity of several different enzymes, all principally associated with the acidic environment within lysosomes ^103^. In dystrophic muscle this stain readily labels the lysosome-rich cytoplasm of infiltrating mononuclear cells (chiefly macrophages) associated with the inflammatory response. More diffuse staining is however also found within the sarcoplasm of degenerating/regenerating myofibres ^70^. Our analysis shows that this stain can indeed be used to discern these pathological features in DE50-MD muscle, and that AP staining can (like PSR) be used as a statistically powerful, age-independent quantitative metric. As with PSR, image analysis of our longitudinal samples suggests a downward trend with increasing age ( Figure 7D). In healthy muscle, where staining appears restricted to capillaries, this can likely be attributed to changes in fibre diameter: capillary density is effectively a perimeter-based metric, and thus will decline proportionally as muscle fibre area increases. Conversely in dystrophic muscle (where the AP fraction is often 10-fold greater) this decline instead reflects fundamental changes in pathology: as shown in the underlying images ( Figure 7B) younger muscle is host to focal infiltrates of densely staining mononuclear cells, suggesting prominent inflammation and macrophage scavenging of necrotic tissue, while older muscle exhibits features more consistent with lower-level fibre degeneration and turnover (note our quantification is area-rather than intensity-based, thus higher AP fractions indicate greater stained tissue area regardless of intensity). A parallel study measuring serum cytokines (manuscript under review) in our natural history cohort revealed high levels of the inflammatory marker CCL2 in DE50-MD animals, which was most prominently elevated in younger samples (<12 months). Our data suggests a peak in inflammation occurs between 3 and 9 months, when animals are actively growing, a correlation that supports the position that dystrophic damage is most severe during muscle growth. These findings are further corroborated by our post-mortem analysis ( Figure 12): samples from younger dystrophic animals (5–7 months, which would fall within the ‘peak’ phase) typically exhibited prominent and intense focal staining consistent with necrosis and infiltrating inflammatory mononuclear cells, while older muscle samples were chiefly host to diffuse sarcoplasmic staining (also reflected via quantitative image analysis). One exception is the diaphragm: very few focal inflammatory regions were found in younger DE50-MD diaphragm muscles, and diffuse sarcoplasmic staining instead predominated at both ages. This, alongside the prominent fibre splitting and hypertrophy, suggests pathology in this muscle might proceed differently, with the peak inflammatory/necrotic phase occurring earlier (prior even to our younger post-mortem ages).

As with PSR, our analysis shows that AP staining represents a valuable quantitative metric for disease, and again illustrates the strengths of body-wide analysis: with N=5, post-mortem measurements could detect therapeutic amelioration at the 25% level (in younger animals, with more prominent pathology, only 3 would be required).

### mRNA markers of dystrophic pathology

In addition to our histological analysis, we also measured gene expression in RNA isolated from serial muscle sections, facilitating comparison of histological and gene expression data. Measurement of gene expression via qPCR has many practical advantages: analysis is comparatively rapid, immediately quantitative, and a single prepared sample can be used to determine expression level of multiple targets (saving material, allowing construction of sample archives, and facilitating cross-comparisons and outlier detection). We selected several markers that served to complement our histological findings (collagen I for fibrosis, osteopontin for inflammation), but also investigated markers more challenging to quantify histologically, such as those associated with muscle regeneration ( *MYH3*, *MYH8*), myoblast proliferation and differentiation ( *MEF2C*, *Myf5*), compensatory expression of the autosomal dystrophin paralogue utrophin, and expression of dystrophin itself (which in dystrophic tissue is absent at the protein level, precluding histological measurement).

Embryonic and neonatal myosin heavy chains (encoded by *MHY3* and *MHY8*, respectively) are specialised developmental myosin isoforms expressed within embryonic/foetal muscle, but not in healthy adult tissue ^104^. After muscle damage, however, the regeneration process recapitulates key elements of embryonic development: *MYH3* is expressed within newly formed myotubes, and *MYH8* appears as fusion and growth proceed toward the mature, regenerated myofibre state ^40^. Both are ultimately replaced by expression of one of the ‘mature’ myosin heavy chains once repair is complete, but this turnover is gradual rather than rapid. Expression of *MYH3* and *MYH8* in adult muscle thus signifies damage, but also indicates ongoing repair. In longitudinally obtained *vastus* samples these two myosin heavy chain genes proved to be strong markers of dystrophic pathology ( Figure 9A, B), with 10–100-fold higher expression in DE50-MD samples, and extremely high statistical significance at all ages (with group sizes of 6, our power calculations showed these markers would be capable of detecting effect sizes as small as 25%). In human patients, muscle regeneration ultimately declines as disease progresses ^5^, and even *mdx* mice show reduced regenerative capacity with increasing age ^105^; however, our DE50-MD data suggests skeletal muscle regeneration remains robust and ongoing in this animal model over our study period. Body-wide ( Figure 14), these markers were similarly elevated, with *MYH3* being markedly higher across our entire muscle panel (and *MYH8* increased in most): based on post-mortem data, theoretically even group sizes of 3 would be sufficient to detect 25% improvements. The utility of *MYH3* as a biomarker for regeneration has been noted by others ^106^, and while this mRNA can be detected in our WT samples, levels are sufficiently low to conclude that (in the dog at least) this gene is not meaningfully expressed in healthy muscle. Similarly, we do not detect embryonic myosin in healthy muscle at the protein level ( Figure 3). Expression of *MYH8* is more interesting. Firstly, levels of this mRNA in healthy muscle, while markedly lower than in dystrophic tissue, are nevertheless consistent with meaningful biological contributions: in contrast to data reported for rodents (where post-natal *MYH8* is found only at very low levels ^107^) and humans (where *MYH8* is restricted to muscle spindles in adult tissue ^40^), in dogs this gene appears to be canonically expressed in post-natal muscle. We note that expression declined modestly with age in both healthy and dystrophic muscle (though always markedly elevated in the latter), implying that presence of this mRNA in healthy muscle might reflect youth. Beagles are essentially fully grown at 18 months, but still comparatively young (healthy animals can live 12–15 years ^108^). Complicating this picture, however, our post-mortem data reveals that the diaphragm and tongue represent prominent outliers. In these two muscles specifically, no significant differences were found for *MYH8* expression between any of our post-mortem groups: a consequence of high expression in healthy muscle, rather than lowered expression in dystrophic tissue. To our knowledge, this high, muscle-specific MYH8 expression has not been reported elsewhere, in the dog or other species. Both the diaphragm and tongue also represent notable outliers via other metrics, but why these two muscles should express this neonatal myosin so prominently (~10–100-fold higher than expression in other muscles) is not clear, meriting future investigation. It is also important to note that (for the remaining muscles) expression of *MYH3* and *MYH8* is correlated with damage and repair generally, rather than dystrophic damage specifically: healthy muscle samples excluded from our analysis on grounds of iatrogenic injury exhibited expression comparable with dystrophic muscle (illustrating the benefit of this corroborating histological insight), but such exclusion is predicated on prior knowledge of genotype, and would not be applicable to pre-clinical trials conducted in a blinded fashion. A repeat biopsy strategy thus carries risks, though skilled technique can minimise these risks: we note that even with 6 samples collected per muscle, iatrogenic injury was comparatively rare within our sample collection (only 9 out of 75 samples showed some evidence of damage), and power calculations for *MYH3* and *MYH8* expression using our full dataset showed that inclusion of injured samples did not substantially alter the utility of these markers (see underlying data ^63^).

*MYH3* and *MYH8* are comparatively late, high abundance markers of regeneration, but are not the sole metrics by which this can be assessed. Muscle repair is mediated by a dedicated stem cell population (the satellite cells) which lie adjacent to myofibres: outside the sarcolemma, but beneath the basal lamina ^89, 109, 110^. Under healthy conditions satellite cells are quiescent, but following damage they activate and proliferate, differentiating to become myoblasts which migrate to the site of injury, before aligning and fusing to form new *MYH3*-positive myotubes, and eventually *MYH8*-positive myofibres (as described above). This progression from quiescent stem cell to post-mitotic multinucleate myofibre involves marked changes in transcription, mediated principally by the myogenic basic helix-loop-helix (bHLH) transcription factor family ( *Myf5*, *MRF4*, *MyoD* and *myogenin*) ^111^, and by the myocyte enhancer factor *MEF2C* ^65^: these factors might thus serve as additional biomarkers for muscle regeneration. We chose here to focus on *Myf5* and *MEF2C*: previous pilot work suggested both these factors were upregulated in DE50-MD muscle ^55^, and IHC data showed dystrophic increases in MEF2 at the protein level ( Figure 3A–C). Our longitudinal analysis confirmed that both transcription factors were consistently expressed at higher levels in dystrophic tissue, though increases were more modest than those seen with the myosin heavy chains, particularly for *MEF2C* (~2-fold). This myocyte enhancer is expressed at moderate levels in healthy muscle ^65, 66^ and can also be weakly detected at the protein level ( Figure 3D): regeneration-specific increases in *MEF2C* expression must consequently be measured against this substantial baseline, placing limits on statistical power ( Table 2) and greater susceptibility to confounding effects of iatrogenic damage (extended data, supplementary figure 2 ^43^). Expression of *Myf5*, in contrast, is held to be more transient, expressed in activated satellite cells (and potentially retained in those cells that return to quiescence) ^112– 114^ but subsequently superseded by the more terminal drivers of myogenesis ( *MyoD* and *myogenin*) as differentiation proceeds. Expression of *Myf5* in healthy *vastus* muscle was indeed low (Cq 26–29), and equivalent dystrophic levels were markedly higher, particularly within the first year of life ( Figure 9D). This biomarker also appeared less sensitive to non-dystrophic injury, suggesting that a more transient role in muscle repair might serve to distinguish acute from chronic damage. This promising behaviour prompted us to carry *Myf5* expression forward to post-mortem analysis, but here this marker performed poorly, with many muscles exhibiting only modest differences in expression between healthy and DE50-MD groups, alongside marked variation within groups and individual muscles. This disappointing finding can partly be attributed to differences in sample size (N=3–4 per group for post-mortem, compared to N=9+ for longitudinal *vastus*), but this high variability might also reflect the more limited temporal data in our post-mortem sample set: a marker expressed prominently yet transiently might be well-represented by repeated sampling at 3-month intervals, but with more sparsely distributed time points, expression might be captured at high levels, or not at all, purely through chance. We did not investigate other myogenic transcription factors. *MyoD* and *myogenin* would be logical choices given their prominent roles in terminal commitment and differentiation ^111, 114^, but such investigations would be driven more by curiosity than biomarker evaluation; our data suggests that myogenic transcription factors are of limited diagnostic power for use in pre-clinical trials. Muscle damage and repair is ongoing within DE50-MD muscle even at 18 months of age (as demonstrated by the robust and maintained expression of *MYH3* and *MYH8*), but dystrophic damage is also asynchronous, with any given muscle sample likely host to multiple regions at different stages in the repair process ^115^: under these conditions, markers expressed later but in a sustained manner (like the myosin heavy chains) are likely to be more statistically reliable than those with transient, stage-specific expression profiles (like the myogenic transcription factors).

To corroborate our histological assessment of muscle fibrosis, and to obtain insights into fibrotic progression at the transcriptional level, we measured expression of muscle collagen. The collagen family has many subtypes, of which collagens I and III are the major forms associated with endo- and perimysial linings ^81^. Studies suggest comparable involvement of both collagens with pathological muscle fibrosis ^98^, so we focussed on collagen 1 ( *COL1A1*). Longitudinal *vastus* data suggested that expression was varied, even within groups (likely reflecting the specific composition of connective tissue within individual biopsy samples) but was also significantly higher in DE50-MD muscle overall. However, the utility of this marker was strongly age-dependent, and in a departure from most other metrics, differences between genotypes were most prominent in older samples (12–18 months), where healthy muscle exhibited significantly lower levels of *COL1A1* than dystrophic. At younger ages, differences between genotypes were more modest, principally due to greater expression in healthy muscle: expression in DE50-MD muscle did not change markedly with age. Comparison of older healthy muscle (where *COL1A1* expression is low) with DE50-MD muscle either young or old (where expression remains high) is thus likely to reveal differences, and accordingly measurement of *COL1A1* in our post-mortem dataset ( Figure 14D) indicated this marker was indeed significantly different between healthy and dystrophic groups for most, though not all, muscles (with similar variation to that seen in longitudinal samples). Taken together these findings suggest a strong association of *COL1A1* expression with muscle growth: both healthy and dystrophic animals are still growing rapidly before 9 months of age, and expression at younger ages might thus reflect tissue remodelling associated with fibre hypertrophy (in dystrophic muscle occurring alongside pathological remodelling associated with fibrotic scarring). This is not necessarily unexpected: collagen proteins can be enormously stable, with half-lives measured in years ^116^, and rates of turnover also decline with age ^117^. Established connective tissue (as measured via PSR) will thus readily persist in the absence of ongoing gene expression. At later time points, growth is minimal but pathology continues, and differences in *COL1A1* expression between genotypes might consequently become more prominent. Consistent with this interpretation, collagen expression in healthy samples excluded on grounds of injury was highly variable: comparable to uninjured muscle in some samples, while markedly elevated in others (ostensibly reflecting later and earlier phases of the muscle repair/remodelling process, respectively).

Collagen expression thus serves as a measure of active connective tissue remodelling rather than a metric for total fibrosis. Sample size/power calculations suggested this marker is sufficient to detect changes at the 50% level with realistic group sizes: similar in statistical power to PSR analysis, but in a manner that complements, rather than replicates, measurement of muscle fibrosis histologically.

Acid phosphatase staining suggested a strong inflammatory component to DE50-MD muscle pathology, with a prominent peak within the first year. In a similar approach to that used for PSR/collagen I (above), we supported these investigations at the transcriptional level via measurement of the inflammatory marker *SPP1* (osteopontin), a secretory glycoprotein that can function both as a cytokine and as a component of the ECM, interacting with a host of different cell types, including macrophages and T-cells ^82^. In healthy tissue, *SPP1* is associated chiefly with epithelia, bone and kidney ^118^, but increases in expression are associated with inflammation and tissue remodelling conditions, particularly those of chronic rather than acute nature ^119– 122^. Dystrophic muscle is host to both persistent inflammation and ongoing tissue remodelling ^123^, and levels of *SPP1* are markedly elevated in mouse models, in the GRMD dog model, and in human patients ^83, 84, 124^. This gene is also a disease modifier, indicating that expression of *SPP1* is not simply associated with pathological inflammation but may in fact be a key driver of this process ^125– 127^. Expression in our longitudinal panel confirmed that this marker is dramatically upregulated in skeletal muscle of the DE50-MD dog, with expression being highest between 3 and 9 months of age, consistent with the peak phase of inflammation indicated by AP staining. Levels in healthy muscle were low at all ages and, more pertinently, were also consistently low in healthy muscle samples with evidence of injury. *SPP1* reflects inflammation rather than damage, and although injury of healthy muscle does elicit an inflammatory response, this is comparatively brief, unlike expression of *MYH3* and *MYH8* (which our data suggest is maintained for some months following injury). In body-wide analysis, this marker also performed strongly: levels in all dystrophic muscles (both young and old) were markedly higher than in healthy muscles, including those more challenging to differentiate via other metrics, such as the diaphragm and (especially) the tongue. Again, consistent with a peak inflammatory phase within the first year of life, samples from younger DE50-MD dogs expressed higher levels of *SPP1* than those from older DE50-MD, and here the differences were sufficient to reach statistical significance in some muscles ( *masseter* and *gracilis*), something no other marker achieved. Power calculations suggested that this marker should detect changes at the 50% level longitudinally, and at the 25% level via post-mortem analysis. *MYH3* and *MYH8* are more strongly upregulated and are thus stronger biomarkers in raw statistical terms, but *SPP1* appears to be upregulated in every DE50-MD muscle assessed, and can discern inflammatory pathology from more generalised damage, making this marker particularly valuable.

We also measured expression of utrophin ( *UTRN*), the autosomal paralogue of dystrophin. In healthy muscle utrophin is found at the neuromuscular junction, but is also expressed during muscle regeneration ^85^. At the protein level, utrophin does not carry the full suite of domains found in dystrophin, but it can nevertheless functionally substitute to some extent ^128^. In the *mdx* mouse this gene exhibits compensatory upregulation ^86^, and targeted upregulation of utrophin has further been proposed as a therapeutic approach ^129– 131^. Pilot IHC work in the proband of the DE50-MD mutation showed utrophin at the sarcolemma of some myofibres ^28^, and our gene expression data here suggests that *UTRN* expression in DE50-MD muscle is greater than in healthy muscle, but only modestly so, suggesting that any compensatory changes are similarly modest. While this expression could be investigated further, for the purposes of this study (identifying viable dystrophic biomarkers) the expression of *UTRN* is of insufficient statistical power to be of pre-clinical value.

Finally, we measured expression of dystrophin itself: while the DE50-MD mutation leads to nonsense mediated decay (NMD) of mature dystrophin mRNAs (and thus absence of dystrophin at the protein level), this does not mean expression cannot be measured. Transcribing a full-length dp427 mRNA from the 2.3 megabase dystrophin gene requires 16 hours ^71^, while the NMD checkpoint occurs only upon transcript maturity ^73^. As we showed previously ^56^, provided care is taken not to rely exclusively on polyA-targeted reverse transcription, nascent (partially completed) transcripts can readily be measured: for dystrophin specifically, we have shown that myonuclei are host to multiple nascent transcripts ^47^ and that these immature transcripts represent the bulk of dystrophin mRNA (as also proposed by others ^71, 72^). When quantified as ‘total’ rather than only ‘mature’, dp427 is a gene of relatively high abundance. Moreover, by targeting different regions of the dystrophin transcript (see Figure 8), quantification can be biased in favour of nascent mRNAs (5’ sequence: exons 1:2) or more mature transcripts (3’ sequence, exons 62–64): as only mature DE50-MD dystrophin mRNAs are subject to degradation, this approach allows reductions in transcriptional initiation (i.e. overall reduction in dystrophin expression) to be distinguished from reductions due to NMD. Therapeutic interventions promoting transcript stability (such as skipping of exon 51) should reduce losses to NMD, but efficiency of skipping might be underestimated if overall dp427 transcription is lower in DE50-MD muscle (as has been shown in *mdx* mouse muscle ^47^).

Analysis of exon 62-64 sequence showed clear reductions in mature transcripts longitudinally ( Figure 8) and systemically ( Figure 13), while both datasets also demonstrated that overall levels of transcriptional initiation are highly comparable between healthy and DE50-MD animals, at all ages and between different muscles. While the statistical power of exon 62-64 sequence is modest, the targeted nature of this measurement means it remains a potentially valuable metric for therapeutic evaluation: our data suggests that correction of dp427 mRNA could be detected in DE50-MD muscle, and that despite ongoing muscle degeneration, regeneration and fibrotic replacement, myonuclear commitment to dystrophin transcription remains at levels comparable to those of healthy muscle. Interestingly, parallel studies in the brains of DE50-MD dogs ^37^ did show modest but significant reductions in total dp427 expression. Levels of full-length dystrophin in the brain are markedly lower than in skeletal muscle, however, and dp427 expression is largely restricted to specific brain regions (primarily the hippocampus and Purkinje cells of the cerebellum) which might be selectively lost or modulated in dystrophic animals. Full-length dystrophin in the brain is moreover predominantly dp427c, an isoform transcribed from a different (and thus potentially alternatively regulated) promoter ^2, 132, 133^.

We were also able to detect modest dystrophy-associated increases in dp71: this short dystrophin isoform is not expressed in muscle fibres but is found in many other tissues, including blood vessel endothelia (accounting for the low but non-zero levels found in healthy muscle) ^74^. We have shown an association of this shorter isoform with mitotic cell populations (dp71 requires only ~1 hour to transcribe, whereas the 16 hours needed for dp427 precludes concurrent cell division), and others have shown expression of dp71 in proliferating myoblasts ^75– 77^. This latter population likely explains the increases measured here.

### Dystrophic disease progression in the DE50-MD dog

Collectively, the multiple lines of complementary quantitative analysis shown here allow a consistent timeline of DE50-MD muscle pathology to be constructed: within the *vastus lateralis* muscle our data shows that damage, degeneration and concomitant regeneration is already present at 3 months of age, accompanied by prominent infiltration of mononuclear cells and expression of inflammatory markers. While myofibres in such young animals are collectively too small to exhibit reduction in median MFD, fibre size variation can already be detected. Dystrophic damage (at this stage at least) tends to be highly focal, presenting in patches that can encompass an entire fascicle, where all fibres appear to be at the same approximate stage of degeneration/regeneration. Muscle regeneration is maintained throughout our study period, and remains robust even at 18 months of age, however levels of regenerative markers are highest within the first year of life. The period from 3 to 9 months is also associated with prominent inflammation, with markedly higher levels of acid phosphatase staining and *SPP1* expression than are detected at later time points. Expression of collagen 1 over this period is also higher than at later time points, in both DE50-MD and healthy muscle, suggesting significant muscle tissue remodelling that is not wholly associated with dystrophic pathology. This correlation of animal growth with peak inflammatory and degenerative damage in the DE50-MD dog indicates that growth-associated muscle hypertrophy (and potentially hyperplasia) elicits the most severe challenges for dystrophic muscle. This pattern is reflected more broadly, with our post-mortem data showing that regeneration continues throughout DE50-MD skeletal muscle even at 18 months of age, but that inflammatory and pathological remodelling changes typically also are higher within the first year of life. Overall dystrophic progression within limb muscles appears to be broadly comparable, with *latissimus dorsi* and *intercostalis* also exhibiting similar behaviour. The *caudal sartorius* (CAS) muscle is a notable exception with respect to fibrotic remodelling, however, with profound endomysial and perimysial fibrosis even in younger samples (alongside markedly elevated *COL1A1* expression). Pathology in the *masseter* and *temporalis* masticatory muscles is also prominent: particularly with respect to inflammatory markers at 5–7 months.

Two muscles consistently exhibited aberrant behaviour, however: the diaphragm and the tongue. The diaphragm (DIA) is an outlier in other animal models: in the mouse, this is the only muscle that becomes substantially fibrosed ^23– 25, 100^. The diaphragm is the first muscle to experience severe dystrophic stress: studies in the CXMD _J_ dog showed that the first breath taken after birth (necessary to inflate the lungs against considerable surface tension) results in profound diaphragmatic damage ^134^. The diaphragm is also the only skeletal muscle that is under essentially continuous use. It is perhaps not surprising, therefore, that our data suggests this muscle is more prominently affected than others. As shown in Figure 10, in contrast to other muscles, the DE50-MD diaphragm undergoes progressive hypertrophy: thickening by as much as 4-fold over age-matched healthy diaphragm by 18 months of age. Partly reflecting the marked fibrosis in this muscle, this also reflects true hypertrophy at the myofibre level, with the diaphragm exhibiting widespread fibre splitting even as early as 5–7 months (a phenomenon observed only rarely in other muscles even at 18 months). These pathological changes influence the behaviour of this muscle in our analysis, in several cases paradoxically rendering it less useful rather than more. Furthermore, along with the tongue, this muscle expresses comparatively high levels of *MYH8* even under healthy conditions, precluding use of this gene expression marker in this tissue. Despite these caveats, the diaphragm plays a critical role in human disease, and consequently remains enormously important to therapeutic evaluation. It seems prudent to include this muscle in any future analysis, and accordingly we ensured the diaphragm was included in our panel of muscles used for body-wide power calculations.

**Figure 15.**
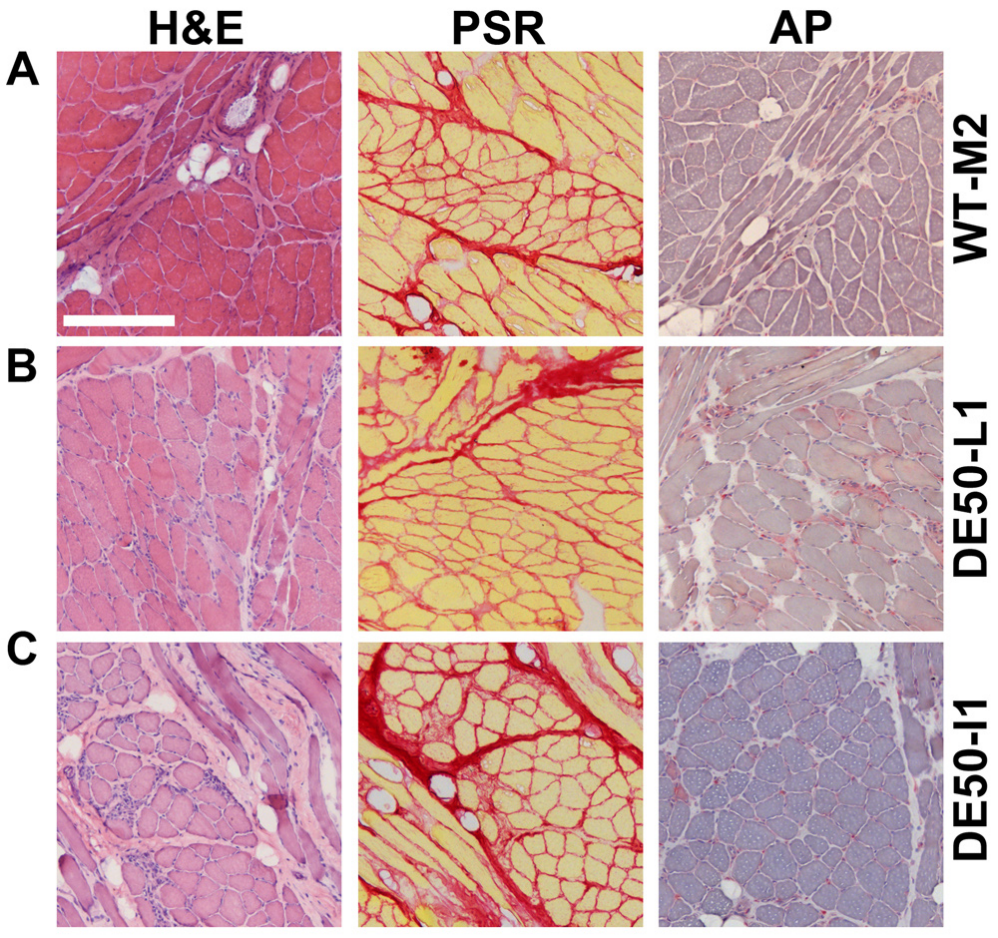
Healthy and dystrophic tongues are histologically comparable. Representative images of histological sections prepared from post-mortem tongue muscle. Sections shown were collected at 18 months of age from ( **A**) one healthy dog (WT-M2), and DE50-MD dogs at ( **B**) 7 months of age (DE50-L1) and ( **C**) 18 months of age (DE50-I1). Sections shown were stained with Haematoxylin and Eosin (H&E), Picrosirius Red (PSR) or Acid phosphatase (AP) as indicated. Healthy tongue muscle contains substantially more connective tissue than other healthy muscles (comparable with dystrophic tongue). Patches of acid phosphatase staining can be seen in DE50-MD muscle, but these are small and diffuse. Scalebar: 200µm.

The tongue (TON) was also an outlier: of all the muscles profiled, this tissue was uniquely ill-suited to assessment of dystrophic pathology. Histologically, PSR and AP staining in the tongue was comparable between groups, as was *COL1A1* and *Myf5* gene expression (though *Myf5* performed poorly in post-mortem tissue overall). *MYH8* in healthy tongue was also expressed at a level comparable to that in dystrophic tissue. In other dog models of DMD, the tongue is frequently reported as one of the more severely affected muscles, associated with macroglossia and concomitant dysphagia ^135– 137^ (also reported in the Cavalier King Charles Spaniel proband for the DE50-MD colony ^28^). Several DE50-MD animals in this study were euthanised due to dysphagia, but macroglossia was not the underlying cause. Quantitatively, DE50-MD tongue muscle is comparable with other dystrophic muscles: the aberrant behaviour of this muscle stems from the fact healthy tongue is also quantitatively comparable with dystrophic muscles ( Figure 15). The perpendicular arrangement of myofibres within the tongue renders this tissue histologically unique ^138^: metrics optimised for more conventional muscles might consequently not be applicable here. Expression of embryonic myosin ( *MYH3*) and osteopontin ( *SPP1*) were however markedly elevated in DE50-MD samples regardless of age, and DE50-MD tongue does exhibit small regions of acid phosphatase staining ( Figure 15B, C), implying that this muscle does indeed experience dystrophic degeneration/regeneration, with concomitant inflammation. 

## Conclusions

The work presented here represents, to our knowledge, the most extensive histological and transcriptional characterisation of dystrophic skeletal muscle pathology ever conducted in a canine model. Our longitudinal *vastus lateralis* collection comprises a total of 144 samples (75 healthy, 69 DE50-MD) spanning 6 time points, each with at least N=9 per group: this well-powered sample set affords a high degree of confidence to age-matched comparisons, and further shows that within healthy and DE50-MD groups of any given age, between-animal variation is minimal for many biomarkers assessed. We supplemented this sample set with a similarly extensive collection of muscles collected post-mortem, comprising 174 samples taken from 17 different muscles, and including both young (5–7 months) and older (18 month) dystrophic animals. Post-mortem analysis allows analysis of multiple muscles, including those impractical to biopsy (such as the diaphragm), providing a body-wide assessment of dystrophic disease progression. Post-mortem collection moreover permits larger samples, potentially offering a more representative assessment of pathology than biopsy: in some muscles this former approach allows the entire tissue transverse section to be analysed. Per-muscle N values for our post-mortem comparisons are more modest (N=3-4) but between-animal data is highly consistent within groups (as seen in our longitudinal data). As shown, applying a repeated measures approach to body-wide data allows assessments to achieve remarkably high statistical power.

This work should also not be taken as exhaustive: we have examined a candidate pool of multiple quantitative metrics and, of those, identified several that robustly distinguish healthy from dystrophic muscle, but detailed exploration of underlying pathological aetiology has not been our focus. This extensive collection of samples (both as histological slides and as cDNA) represents an invaluable resource, however, enabling any future investigations to be conducted rigorously and in considerable depth (indeed this same collection has been used to explore expression of the serum inflammatory biomarker CCL2 (manuscript under review)).

### Pre-clinical trial design using the DE50-MD dog

The principal goal of this work was to identify quantitative histological and gene expression metrics that differentiate healthy and DE50-MD skeletal muscle with high statistical confidence, facilitating assessment of the pre-clinical efficacy of therapeutic interventions: markers capable of detecting even modest ameliorative changes without necessitating large group sizes are consequently particularly valuable. Collectively, our data suggests PSR and AP represent useful histological stains for assessing skeletal muscle pathology in the DE50-MD dog, and that *MYH3*, *MYH8*, *COL1A1* and *SPP1* represent similarly strong biomarkers at the gene expression level (with expression of dystrophin exons 62–64 potentially providing a metric for mature transcript stability). Using these markers in combination, and assessing skeletal muscle both longitudinally (via biopsy) and body-wide (post-mortem) represents a statistically powerful approach. Characterising disease progression in this manner also informs trial design, allowing refinement of future studies to maximise information gain while minimising animal numbers: our data shows that using N=6 per group allows improvements as small as 25% to be detected for most markers. We further show that additional gains in sensitivity typically necessitate impractical group sizes: for the DE50-MD dog, N=6 appears to be not merely sufficient, but also optimal for these muscle-associated metrics.

Our longitudinal data indicates that most significant disease-associated changes in DE50-MD dogs occur within the first year, including the peak inflammatory phase associated with muscle growth. The majority of our quantitative metrics exhibit minimal change beyond 12 months, suggesting that ~1-year trials would be sufficient to demonstrate therapeutic efficacy (we note that *COL1A1* expression is an exception, being of most value in adult animals). Frequency of biopsy could also be reduced: therapeutic amelioration of disease-associated changes could be readily monitored over a 12-month trial via sampling at 4 and 8 months of age (with a 12-month sample collected as a component of post-mortem analysis). Given the consistency of our *vastus* data, repeat sampling of the same muscle is likely unnecessary: sampling the left *vastus* at 4 months, and then the right at 8 months (for example) would eliminate any risk of sampling a previous biopsy site. Sampling could instead be extended to an additional muscle, perhaps from the thoracic limb: as shown by our post-mortem data, the *triceps* exhibits strong disease-associated changes, and (like *vastus*) is an accessible superficial muscle, and of sufficient bulk that biopsy samples can be collected with minimal side effects.

### The DE50-MD dog as a model for human disease

In human DMD patients, initial disease presentation is mild (with the disease not typically diagnosed until 3–6 years of age). Disease in the DE50-MD dog is more severe in this respect, with profound muscle pathology even at 3 months of age (alongside visible muscle atrophy). Similar presentation is found in other canine models (such as the GRMD), and this might thus reflect differences in growth patterns between dogs and humans (our data suggests a strong link between muscle growth and dystrophic damage). The bulk of canine growth occurs in the first year of life, and this growth is rapid ^139^. Human growth is slower (not typically complete until the age of 16–18) but includes two major growth spurts (the first two years of life, and at puberty) ^140^. Despite these differences, dogs remain much closer to humans than mice: not only do mouse models of DMD exhibit mild pathology, growth rates and time-to-maturity in mice are such that therapeutic trials of any substantial duration must necessarily be conducted in adult animals ^141^. The ability to monitor disease progression throughout growth is thus a major advantage offered by dog models. Subsequent muscle growth (and associated fusion of new myonuclei) might potentially ‘dilute’ gene therapies delivered at early ages ^142^, but for human patients, preservation of muscle tissue is key: concerns over dilution effects are rendered moot if muscle cannot be preserved in the first place, and therapeutic interventions will thus ideally occur as early as possible after diagnosis. Our data suggests that skeletal muscle histopathological changes in the DE50-MD dog are comparable to those seen in young DMD patients, spanning the period prior to loss of ambulation, but at or beyond the point at which muscle atrophy and weakness become evident: exactly the timeframe over which therapies are most likely to be employed, and over which any clinically beneficial therapeutic effects will be most readily detected. The DE50-MD dog is thus an ideal pre-clinical model for evaluation of therapeutics aiming to abrogate or ameliorate dystrophic progression.

## Data Availability

All data used in this manuscript is available at the figshare repository within the collection “Skeletal muscle phenotype of the DE50-MD dog model -project collection”. https://doi.org/10.6084/m9.figshare.c.6182161.v2 ^143^ Figshare: Skeletal muscle phenotype of the DE50-MD dog: H&E https://doi.org/10.6084/m9.figshare.20456913.v1 ^50^ This dataset contains all the skeletal muscle haematoxylin and eosin images shown in this manuscript ( Figure 2, Figure 10 and Figure 15), along with all additional images used for qualitative assessment of pathological features in longitudinal (vastus lateralis) and post-mortem samples (multiple muscles), including diaphragm thickness data shown in Figure 10. figshare: Skeletal muscle phenotype of the DE50-MD dog: immunofluorescence https://doi.org/10.6084/m9.figshare.20473593.v1 ^52^ This dataset contains all the skeletal muscle immunofluorescence images shown in Figure 3, along with all additional images used for qualitative assessment of pathological features. figshare: Skeletal muscle phenotype of the DE50-MD dog: fibre profiles and revertants https://doi.org/10.6084/m9.figshare.20473512.v1 ^51^ This dataset contains all the images used for calculation of minimum Feret diameter (MFD) distributions, medians, and coefficients of variation shown in this manuscript ( Figure 4) as well as those used for evaluation of revertant fibres ( Figure 5). Raw per-image data and a summary spreadsheet are also included. figshare: Skeletal muscle phenotype of the DE50-MD dog: picrosirius red https://doi.org/10.6084/m9.figshare.20436099.v2 ^48^ This dataset contains all the images used for evaluation and quantification of muscle fibrosis via picrosirius red staining, and all derived per-image and per-sample fibrosis fraction values, along with a spreadsheet sample key. figshare: Skeletal muscle phenotype of the DE50-MD dog: acid phosphatase https://doi.org/10.6084/m9.figshare.20439804.v2 ^49^ This dataset contains all the images used for evaluation and quantification of muscle inflammation via acid phosphatase staining, and all derived per-image and per-sample AP fraction values, along with a spreadsheet sample key. figshare: Skeletal muscle phenotype of the DE50-MD dog: qPCR data https://doi.org/10.6084/m9.figshare.20466957.v2 ^59^ This dataset contains two excel spreadsheets (one for longitudinal vastus, one for post-mortem) containing all underlying qPCR data (raw Cq values, normalised data and log transformed values used for statistical analysis and Figure 8, Figure 9, Figure 13 and Figure 14), along with exported output data from SPSS mixed-model analysis and Holm-Sidak correction analysis. figshare: Skeletal muscle phenotype of the DE50-MD dog: SPSS data https://doi.org/10.6084/m9.figshare.20468544.v2 ^61^ This dataset contains three SPSS data documents (longitudinal vastus with iatrogenic damage samples excluded, longitudinal vastus with iatrogenic samples included, and post-mortem), each containing all underlying data for the quantitative metrics assessed in this manuscript. This dataset also contains all individual data output files for each mixed-model analysis and post-hoc multiple comparisons, and a Graphpad prism (.pzfx) file containing all Holm-Sidak multiple-comparisons correction analysis. figshare: Skeletal muscle phenotype of the DE50-MD dog: PRISM data https://doi.org/10.6084/m9.figshare.20468613.v2 ^60^ This dataset contains Graphpad prism files used to generate the figures shown in this manuscript ( Figure 4, Figure 5, Figure 6, Figure 7, Figure 8, Figure 9, Figure 11, Figure 12, Figure 13 and Figure 14), and the exported graphs themselves. figshare: Skeletal muscle phenotype of the DE50-MD dog: GLIMMPSE data https://doi.org/10.6084/m9.figshare.20468916.v2 ^63^ This dataset contains the GLIMMPSE input files (.json format) and exported GLIMMPSE analysis (as excel files) for longitudinal vastus lateralis and post-mortem muscle panel sample size/power calculations (as shown in Table 2 and Table 3). A summary file is also provided as an excel spreadsheet. figshare: Skeletal muscle phenotype of the DE50-MD dog: extended data https://doi.org/10.6084/m9.figshare.20473548.v1 ^43^ This dataset contains the following supplementary figures: Supplementary figure 1: Samples from DE50-MD animals euthanised early are similar to age-matched samples from DE50-MD animals euthanised late Supplementary figure 2: Gene expression analysis including WT samples with evidence of iatrogenic damage Data are available under the terms of the Creative Commons Attribution 4.0 International license (CC-BY 4.0). figshare: ARRIVE checklist for ‘The skeletal muscle phenotype of the DE50-MD dog model of Duchenne muscular dystrophy’. https://doi.org/10.6084/m9.figshare.20971714.v1 ^144^
