# Supplementary material for: The skeletal muscle phenotype of the DE50-MD dog model of Duchenne muscular dystrophy
Source: Wellcome Open Res. 2022 Sep 23;7:238. [Version 1] doi: 10.12688/wellcomeopenres.18251.1 (PMC9971692; doi:10.12688/wellcomeopenres.18251.1)
Supplement: Peer review history

# Peer review: Reviewer response for version 1

**DOI:** 10.21956/wellcomeopenres.20230.r54134

**Published:** 2023-02-07

**Authors:** Andrés G. Vidal-Gadea

**Affiliations:** 1 School of Biological Sciences, Illinois State University, Normal, IL, USA

The field of Duchenne muscular dystrophy (DMD) research has benefitted from the contribution of several model organisms ranging from zebrafish and mice to flies, worm, and dogs. While dogs are not genetically amenable, are expensive to maintain, and slow to grow, they also provide one of the closest matches to DMD in terms of the severity of the phenotypes caused by the disease when compared with just about any other model of this disease. In the past several groups have established dystrophic canine colonies with mixed results. In the present manuscript, Hildyard and colleagues provide the most in-depth natural history study of any animal modeling DMD (if memory serves). In it, they conduct a longitudinal analysis of the *Vastus lateralis* (one of the muscles to show greatest dystrophic damage) over an 18-month period and supplemented this with extensive postmortem analysis of other muscles. Muscle biopsies were used for qPCR analysis and for histology using standard techniques. The authors further produced a thorough statistical power analysis of their data in order to establish the minimal sample sizes required for different types of investigations and effect sizes. Lastly, a thoughtful guideline for animal care, handling, and euthanasia was described to help standardize future studies.

 In my opinion this is a foundational study that is part of a larger effort to establish the DE50-MD canine strain as a go-to model for Duchenne muscular dystrophy studies. This will be particularly impactful for preclinical testing treatments such as exon skipping and other drugs for which murine and other animal models are often unreliable surrogates. Additionally, the extent and the consistency of the degeneration seen in all the dystrophic animals exceeded that seen with other canine models. This makes the work all the more exciting.

 The study really is a tour de force in the sheer number of experiments and data presented. I will not criticize this study needlessly, but I will say that one suggestion I have was with regards to sampling. Because there is so much extensive data now on the relative pathophysiological status of different muscles in these animals, it would have been great if biological samples associated with them had been preserved in Trizol (or other media) for future transcriptomic analysis beyond that undertaken in this study. Overall, a very well planned, executed, and written study.

Is the work clearly and accurately presented and does it cite the current literature?

Yes

If applicable, is the statistical analysis and its interpretation appropriate?

Yes

Are all the source data underlying the results available to ensure full reproducibility?

Yes

Is the study design appropriate and is the work technically sound?

Yes

Are the conclusions drawn adequately supported by the results?

Yes

Are sufficient details of methods and analysis provided to allow replication by others?

Yes

Reviewer Expertise:

Muscle physiology, neuroscience, Duchenne

I confirm that I have read this submission and believe that I have an appropriate level of expertise to confirm that it is of an acceptable scientific standard.

# Peer review: Reviewer response for version 1

**DOI:** 10.21956/wellcomeopenres.20230.r53296

**Published:** 2022-11-30

**Authors:** Alyson A. Fiorillo, Nikki McCormack

**Affiliations:** 1 Center for Genetic Medicine Research, Children’s Research Institute, Children’s National Hospital, Washington, DC, USA; 2 The George Washington University School of Medicine and Health Sciences, Washington, DC, USA; 3 Center for Genetic Medicine Research, Children’s Research Institute, Children’s National Hospital, Washington, DC, USA

Summary:

 The manuscript by Hildyard *et al.* provides a comprehensive of skeletal muscle in the DE50-MD dog model of DMD. The authors used muscle biopsies from DE50-MD dogs at multiple timepoints to assess histology (inflammation, fibrosis, myofiber size, revertant dystrophin) and gene expression (regeneration, dystrophin, collagen, inflammation) in the vastus lateralis. The authors also analyzed these metrics in 17 different skeletal muscles post-mortem. The data in this study will help inform future pre-clinical trials using this model as it provides histological and transcriptional biomarkers and sample sizes necessary for detecting statistical differences.

Minor comments:

1. Add labels to the sample key in Figure 6-9.
2. Figure 9: add utrophin staining of vastus lateralis to complement gene expression data.
3. Figure 15 should be introduced in the results section.
4. More details about how fiber splitting (Fig 4) was assessed would be helpful. Since the authors did not isolate individual fibers, it is unclear how splitting can be properly assessed. 

This is the most thorough assessment to date of the DE50-MD dog model and with be extremely useful to the DMD field.

Is the work clearly and accurately presented and does it cite the current literature?

Yes

If applicable, is the statistical analysis and its interpretation appropriate?

Yes

Are all the source data underlying the results available to ensure full reproducibility?

Yes

Is the study design appropriate and is the work technically sound?

Yes

Are the conclusions drawn adequately supported by the results?

Yes

Are sufficient details of methods and analysis provided to allow replication by others?

Yes

Reviewer Expertise:

NA

We confirm that we have read this submission and believe that we have an appropriate level of expertise to confirm that it is of an acceptable scientific standard.
